# The RNA Chaperone Hfq Is Essential for Virulence and Modulates the Expression of Four Adhesins in *Yersinia enterocolitica*

**DOI:** 10.1038/srep29275

**Published:** 2016-07-08

**Authors:** Tamara Katharina Kakoschke, Sara Carina Kakoschke, Catharina Zeuzem, Hicham Bouabe, Kristin Adler, Jürgen Heesemann, Ombeline Rossier

**Affiliations:** 1 Max von Pettenkofer Institute for Hygiene and Medical Microbiology, Ludwig Maximilians University, Pettenkoferstrasse 9a, 80336 Munich, Germany

## Abstract

In *Enterobacteriaceae*, the RNA chaperone Hfq mediates the interaction of small RNAs with target mRNAs, thereby modulating transcript stability and translation. This post-transcriptional control helps bacteria adapt quickly to changing environmental conditions. Our previous mutational analysis showed that Hfq is involved in metabolism and stress survival in the enteropathogen *Yersinia enterocolitica*. In this study we demonstrate that Hfq is essential for virulence in mice and influences production of surface pathogenicity factors, in particular lipopolysaccharide and adhesins mediating interaction with host tissue. Hfq inhibited the production of Ail, the Ail-like protein OmpX and the MyfA pilin post-transcriptionally. In contrast Hfq promoted production of two major autotransporter adhesins YadA and InvA. While protein secretion *in vitro* was not affected, *hfq* mutants exhibited decreased protein translocation by the type III secretion system into host cells, consistent with decreased production of YadA and InvA. The influence of Hfq on YadA resulted from a complex interplay of transcriptional, post-transcriptional and likely post-translational effects. Hfq regulated *invA* by modulating the expression of the transcriptional regulators *rovA*, *phoP* and *ompR*. Therefore, Hfq is a global coordinator of surface virulence determinants in *Y. enterocolitica* suggesting that it constitutes an attractive target for developing new antimicrobial strategies.

Quick adaptation to changing environmental conditions is key to a pathogen’s success in the infected host. To promote host colonization and invasion, pathogenic bacteria express specialized surface proteins such as adhesins but also remodel their surface to resist host defence mechanisms. The Gram-negative bacterium *Yersinia enterocolitica* along with *Y. pseudotuberculosis* causes gastrointestinal infections in humans and warm-blooded animals. Upon ingestion of contaminated food or water, enteropathogenic yersiniae cross the intestinal barrier preferably through the M-cells of the terminal ileum and multiply mainly extracellularly in the underlying lymphatic tissue, called Peyer’s patches. They eventually disseminate to mesenteric lymph nodes and conditionally into the spleen and liver^1^. Enteropathogenic yersiniae possess several proteins exposed at their surface that are crucial for virulence in the mouse model of yersiniosis. They include three non-fimbrial adhesins InvA, YadA and Ail which promote adherence to host cells^2^,^3^. InvA (or Inv) constitutes the major intestinal invasin and plays an essential role in bacterial transcytosis across the epithelium^2^,^3^. In addition to their role in binding eukaryotic cells through extracellular matrix proteins, Ail and YadA are key players in resistance to complement^2^,^3^,^4^. Also associated with the bacterial envelope is the type III secretion system (T3SS) Ysc, a sophisticated machinery that injects several Yop anti-host proteins into the host cell cytosol. Yop proteins collectively inhibit phagocytosis and dampen the inflammatory response. The *yop* and *ysc* genes are carried on the virulence plasmid pYV, together with the gene encoding YadA^5^. Finally, the lipopolysaccharide (LPS) also promotes virulence of *Y. enterocolitica* in mice as demonstrated by the attenuation of strains with LPS lacking O-antigen (O-Ag)^6^,^7^ and strains mutated in the lipid A 3′-O-deacylase LpxR/SpfA^8^,^9^.

*Y*. *enterocolitica* exhibits many traits that respond to the temperature encountered during infection of a mammalian host. Upon a switch from 27 °C (optimal growth temperature) to 37 °C (host temperature), the bacteria remodel their surface: (i) they downregulate production of flagella, InvA, and the transfer of O-Ag onto the LPS^10^,^11^,^12^ and (ii) they increase production of YadA, Ail, Ysc T3SS and the LpxR-mediated LPS modifications^4^,^8^,^9^,^13^,^14^. Another surface appendage, the Myf fimbriae, is upregulated upon growth at 37 °C at acidic pH^15^. Although the role of Myf in *Y*. *enterocolitica* pathogenicity has so far not been studied, it is expressed during human infection and can be used for serological diagnostic of yersiniosis^16^. A pilus homologous to Myf, called pH6 antigen (Psa), is produced by *Y*. *pseudotuberculosis* and the plague pathogen *Y*. *pestis*, where it promotes adhesion to host cells and/or resistance to phagocytosis and virulence^2^,^3^.

The deployment of surface virulence factors is tightly controlled in yersiniae. For example, activation of early virulence genes, such as *invA*, is controlled by RovA, a dimeric winged helix transcriptional regulator. At 27 °C, RovA alleviates transcriptional repression by the histone-like protein H-NS through binding to the promoter regions of several genes, including *invA* and *rovA* itself^12^,^17^. A temperature switch to 37 °C leads to reduced DNA binding and increased proteolysis of RovA in *Y*. *enterocolitica* strains of serotype O:8^18^. In contrast virulence genes required for extraintestinal survival, such as *yadA* or a subset of *ysc* genes, are activated at 37 °C by VirF, an AraC-type transcriptional regulator encoded by the pYV plasmid^5^,^14^. Several two-component regulatory systems (TCS) also control expression of surface pathogenicity factors upon sensing different stimuli, e.g. the EnvZ/OmpR TCS confers resistance to high osmolarity, low pH, oxidative stress or complement^19^,^20^ and downregulates the expression of adhesin genes *invA*, *ail* and *yadA*^20^,^21^,^22^ while activating flagellar genes^23^. Other examples include the TCS PhoP/PhoQ and PmrA/PmrB, which together with RovA, govern genes involved in LPS modifications^8^,^24^.

The past decade has seen an increased interest in post-transcriptional regulatory mechanisms mediated by small RNAs (sRNAs). These regulators, usually 50–250 nucleotides in length, interact with mRNA through short imperfect base-pairing and thereby modulate mRNA stability and translation^25^. While fine-tuning protein synthesis, the involvement of sRNAs in bacterial regulatory networks is predicted to provide faster responses and modulate cell-to-cell variability^26^. Therefore, sRNA-mediated regulation is likely to allow bacteria to adapt quickly to changes in the environment. In enterobacteria, a major class of sRNAs require the RNA chaperone Hfq for their stability and function^27^. First discovered as a host factor essential for the replication of the RNA bacteriophage Qβ in *Escherichia coli*, Hfq is a widely conserved protein that stabilizes sRNAs, facilitates sRNA-mRNA pairing and modulates the degradation of target RNAs^27^. In *Salmonella enterica* serovar Typhimurium Hfq impacts the expression of 10–20% genes in the genome and interacts with 30% of the identified Hfq-dependent transcripts^28^,^29^, thereby constituting a global regulatory hub.

In *Yersinia*, Hfq is also likely to be a major regulator. In *Y*. *pestis* a mutation in *hfq* leads to instability of more than a dozen of sRNAs^30^,^31^ and changes in the abundance of 6% of mRNAs, mostly involved in stress responses or metabolism^32^. Also in *Y. pestis*, Hfq exerts its action post-transcriptionally to repress expression of the guanylate cyclase HmsT and to promote expression of cAMP receptor protein Crp and the T3SS effector YopJ, which all contribute to pathogenicity^33^,^34^,^35^. In all three human pathogenic *Yersinia* species, Hfq confers resistance to stress^32^,^36^,^37^ and at least in *Y*. *enterocolitica* and *Y*. *pestis*, Hfq is involved in bacterial metabolism and growth^34^,^37^. In *Y*. *enterocolitica*, we have shown that Hfq is also involved in the production of virulence factors with the upregulation of urease, which protects bacteria from acidic pH, and downregulation of the siderophore yersiniabactin, an iron scavenger^37^. Moreover, our proteomic study suggested that Hfq influences the composition of the outer membrane of *Y. enterocolitica* by repressing the production of several outer membrane proteins (OMPs), i.e. LpxR, the siderophore receptors FyuA and FcuA, and the Ail-like protein OmpX^37^. Significantly, both in *Y*. *pestis* and *Y*. *pseudotuberculosis*, Hfq promoted the production of most Yop effectors and was essential for virulence in mice^32^,^34^,^36^. In contrast, we have demonstrated for two strains of *Y*. *enterocolitica* that Hfq is dispensable for production or secretion of T3SS effectors in response to low-calcium concentration in a host-free system^37^, suggesting that Hfq has a different function among yersiniae. A recent analysis of the genus *Yersinia* indicated that *Y*. *enterocolitica* has acquired the virulence plasmid pYV and the adhesin gene *ail* through an independent and parallel evolutionary path from that of *Y*. *pseudotuberculosis* and *Y*. *pestis*^38^. This different evolution might have resulted in a distinct role for Hfq in the regulation of pathogenicity factors between the pathogenic *Yersinia* spp.

In this study, following on our proteomic analysis that uncovered changes in OMPs, we investigated the role of Hfq in the expression of surface pathogenicity factors, in particular adhesins mediating interaction of *Y*. *enterocolitica* with host cells. Moreover, we were interested in testing whether Hfq plays a role in virulence of *Y*. *enterocolitica* despite being dispensable for T3SS. Using immunoblotting and reporter fusions, we dissected the impact of the RNA chaperone for gene expression at the transcriptional and post-transcriptional levels.

## Results and Discussion

### Increase in production of the adhesin Ail and the Ail-like protein OmpX in the absence of Hfq

In this study we used strains of *Y*. *enterocolitica* serotype O:8 from two different lineages^39^ with very similar results. In the interest of clarity we will mainly present results obtained with parent strain JB580v (an 8081-derivative) and its *hfq* mutant SOR17, except when more data were generated with strain WA-314 and its *hfq*-negative derivative SOR4.

**Figure 1:**
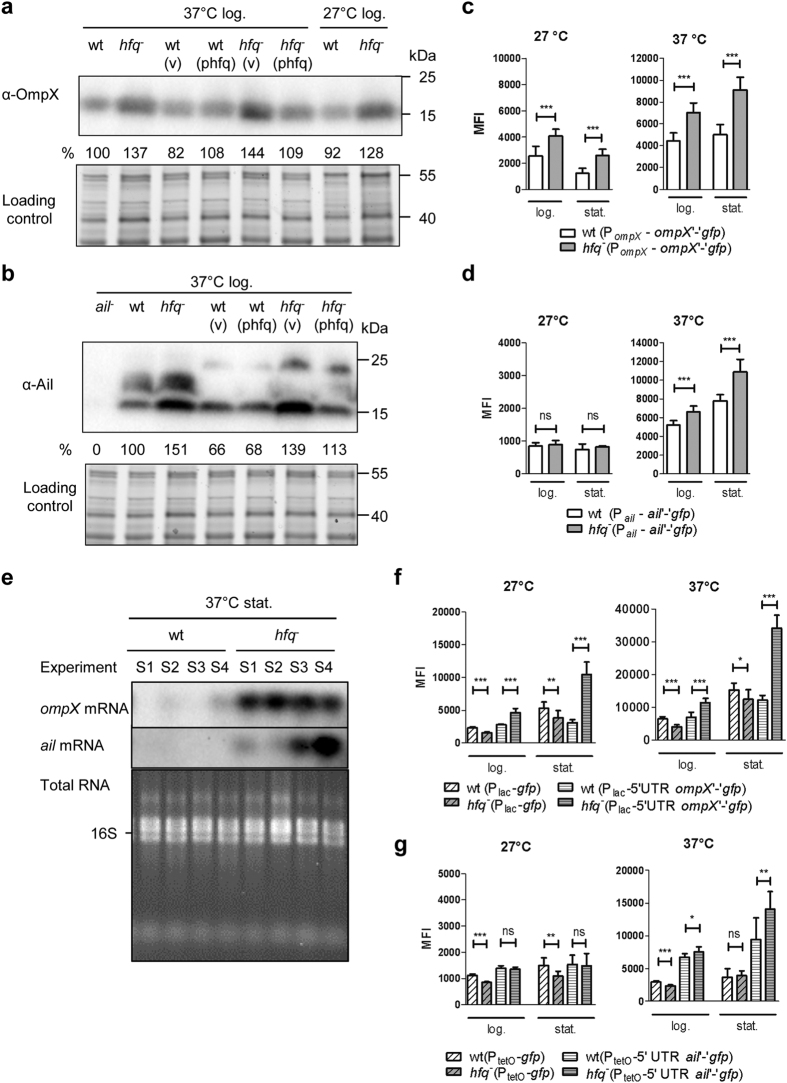
Hfq inhibits *ompX* and *ail* expression at the post-transcriptional level. **(a,b**) Immunodetection of OmpX (**a**) and Ail (**b**) in *Y*. *enterocolitica* parental strain JB580v and *hfq* mutant SOR17 grown for 4 h in LB. Upper panels show immunoblots, and lower panels show part of the gels stained for total proteins. (v), vector pACYC184ts; (phfq), plasmid phfq. Percentages indicate the relative signal intensities: the signal was first normalized relative to total proteins loaded and then compared to that of the normalized signal for the parental strain. In (**b**) the monoclonal antibody reacts with three bands that are absent in the *ail* mutant (lane 1) and therefore correspond to different forms of Ail. In strains carrying plasmids (lanes 4–7), the larger Ail forms are less abundant and increase their apparent molecular weight. For our semi-quantitative analysis, we pooled the normalized signal for all bands. **(c,d,f,g**) Fluorescence of strain JB580v and derivatives carrying translational fusions with *gfp* was measured by flow cytometry upon growth in LB for 4 h (log.) and 22 h (stat.). Results are the mean fluorescence intensity (MFI) and standard deviation of at least three independent experiments, each with three independent cultures per strain. Significance was calculated with Student’s unpaired *t*-test (***P ≤ 0.001; **P ≤ 0.01; *P ≤ 0.05; ns, not significant *P* > 0.05). In these experiments, the MFI of strains carrying a promoter-less *gfp* gene was below 25 units. MFI of strains carrying: **(c)** *ompX*‘-’*gfp* fusion under control of P_ompX_ promoter, **(d**) *ail*‘-’*gfp* fusion under control of P_ail_ promoter, **(f)** plasmids pFX-1 (*gfp* under control of P_lac_ promoter) and pFX-Plac-ompX (*ompX* 5′UTR and *ompX*‘-’*gfp* fusion under control of P_lac_) and **(g)** pFX-2 (*gfp* under control of P_LtetO-1_ promoter) and pFX-PtetO-ail (*ail* 5′UTR and *ail*‘-’*gfp* fusion under control of P_LtetO-1_). **(e)** Northern blotting with DIG-labelled *ompX*- or *ail*-specific RNA probe. Bacteria were grown on four separate occasions in LB at 37 °C for 17 h (S1–S4). Upper two panels: Northern blots and bottom panel: ethidium-bromide stained total RNA. Due to an intervening sequence, the 23S rRNA is processed into two species that flank the 16S rRNA.

Since our previous proteomic analysis suggested that Hfq represses the production of several OMPs, including OmpX^37^, we aimed to confirm this finding by immunoblotting. Using an antibody specific for *E*. *coli* OmpX^40^, we could detect a single band with the expected size in extracts of *Y*. *enterocolitica* (Fig. 1a). Loss of *hfq* led to a ca. 40% increase in OmpX, a phenotype that could be complemented with plasmid phfq which carries the *hfq* gene (Fig. 1a). Our results indicate that, similarly to what has been observed in *E. coli* and *S*. Typhimurium^41^,^42^, Hfq represses production of OmpX in *Y*. *enterocolitica*. Although the function of OmpX is not characterized in *Y*. *enterocolitica*, Ail, a protein with 37% identity and 56% similarity to OmpX is a well-known virulence factor involved in attachment and serum resistance^4^,^13^. Therefore, we also tested whether *hfq* influences the production of Ail. Using a monoclonal antibody^4^, we could detect three Ail-specific bands of ca. 15, 20 and 21 kDa in *Y. enterocolitica* total extracts (Fig. 1b). When grown at 37 °C, temperature for maximal *ail* expression, the *hfq* mutant produced more Ail than their isogenic parents, and introduction of plasmid phfq into the mutants reversed the observed accumulation (Fig. 1b). Hence, *hfq* negatively regulates production of OmpX and Ail in *Y*. *enterocolitica*.

### Post-transcriptional regulation of OmpX and Ail depends on Hfq

Next to determine whether expression of *ompX* and *ail* was controlled by Hfq, we generated plasmids carrying translational fusions with the gene encoding the green fluorescent protein (*gfp*). Plasmids pFX-ompX and pFX-ail include the gene promoter, 5′ UTR and the first 5 or 9 codons of *ompX* or *ail*, respectively, fused in frame with the coding sequence of *gfp*. With these constructs, alterations in fluorescence are likely to reflect changes resulting from both transcriptional and translational control of the genes of interest. Fluorescence intensity of individual bacteria was measured by flow cytometry. As a control for background fluorescence, we used strains harbouring pFX-0, a plasmid with a promoter-less *gfp* gene^43^ (data not shown). Upon growth at 27 °C and 37 °C, the *hfq* mutant carrying *ompX*‘-’*gfp* fusion had a ca. 2-fold increase in mean fluorescence intensity (MFI) in stationary phase compared to the parent (Fig. 1c), supporting the notion that Hfq represses expression of *ompX* in *Y*. *enterocolitica*. With the fusion *ail*‘-’*gfp*, we also observed a slight increase in MFI (ca. 30%) for the *hfq* mutant compared to the parent at 37 °C, also in stationary phase (Fig. 1d). To further investigate the influence of Hfq on *ompX* and *ail* expression we assessed transcript abundance in parent JB580v and *hfq* mutant SOR17 by Northern blotting (Fig. 1e). RNA was isolated in four independent experiments done at 37 °C. In stationary phase, *ompX* and *ail* mRNAs were barely detectable in the parent, but strikingly more abundant in the *hfq* mutant (Fig. 1e) (even if *ail* mRNA amounts varied between experiments). These results are consistent with those obtained with the translational fusions. In log phase at 37 °C, the abundance of *ompX* and *ail* transcripts differed greatly between independent experiments despite identical experimental conditions: no significant difference in mRNA abundance between wild type and *hfq* mutant could be detected, either by Northern blotting or by RT-qPCR performed in three additional and independent experiments (Supplementary Fig. S1). Taken together, our results indicate that Hfq represses expression of *ompX* and *ail* in stationary phase.

Since the direct effects mediated by Hfq-dependent sRNAs typically occur within the 5′ UTR or first codons of the regulated mRNAs (including *ompX* in *S*. Typhimurium^41^), we tested whether the repression of *ompX* and *ail* expression was post-transcriptional. First, we generated plasmid pFX-Plac-ompX, where the promoter of *ompX* was replaced by the P_lac_ promoter. In this plasmid P_lac_ controls expression of the 5′ UTR of *ompX* and the first five codons of *ompX* fused to *gfp*. As a control we used plasmid pFX-1, which carries *gfp* under the control of the P_lac_ promoter^43^. As seen in Fig. 1f, although the P_lac_ promoter was less active in the *hfq* mutant than in the parent (with a decrease in MFI up to 40%), the MFI of the *hfq* mutant carrying pFX-Plac-ompX was increased by 200% in stationary phase at both temperatures (Fig. 1f). Second, we generated plasmid pFX-PtetO-ail, where the promoter P_LtetO-1_^44^ controls expression of the 5′ UTR and first nine codons of *ail* fused to *gfp*. As a control we used pFX-2, which carries *gfp* under the control of P_LtetO-1_. Similar to P_*lac*_, P_LtetO-1_ activity was slightly decreased in the absence of *hfq* (Fig. 1g). In contrast, in the *hfq* mutant carrying pFX-PtetO-ail, fluorescence was increased by 50% relative to the parent upon growth to stationary phase at 37 °C (Fig. 1g). Thus Hfq inhibits expression of *ompX* and *ail* post-transcriptionally, mostly in stationary phase. With the results from the Northern blotting (Fig. 1e), our analysis suggest that Hfq inhibits the stability of *ompX* and *ail* transcript in stationary phase in *Y*. *enterocolitica*. This hypothesis could so far not be tested further, as both transcripts are almost undetectable in the parent strain in stationary phase.

### Hfq-dependent expression of chromosome-encoded invasin

**Figure 2:**
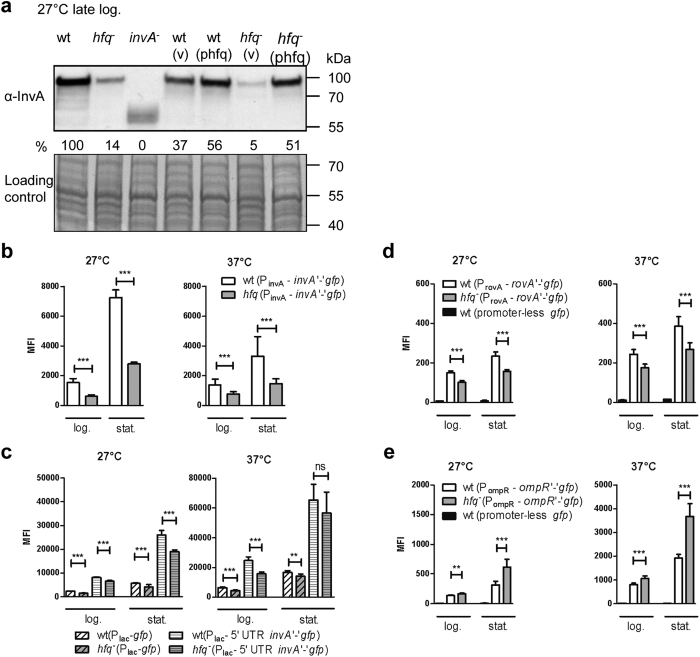
Hfq promotes expression of invasin and exerts opposite effects on expression of the regulatory genes *rovA* and *ompR*. **(a)** Immunodetection of InvA in *Y*. *enterocolitica* strain JB580v and derivatives. Bacteria were grown for 6 h in LB at 27 °C. Upper panel shows the immunoblot and bottom panel shows part of the Coomassie blue-stained gel used as loading control. (v), vector pACYC184; (phfq), plasmid phfq. The *invA* mutant produces a truncated InvA protein. The relative signal for full-length InvA compared to the parental strain (which was set to 100%) is indicated. **(b,c)** Fluorescence of isogenic strains of JB580v carrying, in (**b**): plasmid pFX-invA (translational fusion of *invA* with *gfp* under the control of P_invA_ promoter), and in (**c**): plasmid pFX-1 (*gfp* under control of P_lac_ promoter) or pFX-Plac-invA (*invA* 5′UTR and *invA*‘-’*gfp* fusion under control of P_lac_ promoter). Bacteria were grown in LB for 4 h (log.) and 22 h (stat.) and fluorescence was measured by flow cytometry. Results are the MFI and standard deviation of three (**b**) or two (**c**) independent experiments, each with three independent cultures per strain. **(d,e)** Fluorescence of parental strain JB580v and its isogenic *hfq* mutant carrying translational fusions of *rovA* (**d**) and *ompR* (**e**) with *gfp* was measured by flow cytometry. Bacteria carrying a promoter-less *gfp* (on plasmid pFX-0) were used to measure background fluorescence. Results are the MFI of at least two independent experiments, each with three independent cultures per strain. Significance was calculated with Student’s unpaired *t*-test (***P ≤ 0.001; **P ≤ 0.01; ns, not significant, *P* > 0.05).

As a next step in our analysis, we tested whether *hfq* influenced production of additional adhesins encoded in the chromosome of *Y*. *enterocolitica*, such as InvA. At 27 °C, temperature known for maximal *invA* expression, immunoblotting revealed a ca. 80-% reduction in InvA produced by the *hfq* mutant compared to the parent, which could be complemented with plasmid phfq (Fig. 2a), indicating that Hfq promotes production of InvA in *Y*. *enterocolitica*. We further assessed whether Hfq had an influence on gene expression using a translational fusion of the first four codons of *invA* with *gfp* under the control of the promoter of *invA* (on plasmid pFX-invA). We observed a 2- to 3-fold decrease in MFI for the *hfq*-negative strain relative to the parent at 27 °C and 37 °C (Fig. 2b), indicating that Hfq facilitates the expression of *invA*. To examine whether Hfq exerts its effect at the post-transcriptional level, we generated plasmid pFX-Plac-invA, where the 5′-UTR and first four codon of *invA* are under the control of the P_lac_ promoter. The *hfq* mutant carrying this plasmid exhibited a ca. 15–35% reduction in MFI compared to the parent (Fig. 2C). This decrease was not greater than the reduction in MFI observed with the control plasmid pFX-1 (Fig. 2c), suggesting that Hfq might mainly promote transcription of *invA*.

Expression of invasin is controlled by several transcriptional regulators, including the activator RovA and the repressor OmpR which both interact directly with the promoter region of *invA*^17^,^21^. To test whether Hfq influences expression of both regulatory genes, we used plasmids pFX-rovA and pFX-ompR, which carry the promoter, 5′UTR and the first six or four codons of *rovA* or *ompR*, respectively, fused to *gfp*. Compared to the parent strain, the *hfq* mutant expressed slightly less *rovA* (30%) but 2-fold more *ompR* in stationary phase (Fig. 2d,e). Thus our results suggest that Hfq positively impacts *invA* expression by promoting expression of the transcriptional activator RovA (albeit modestly) and inhibiting expression of the repressor OmpR. Although we only tested a gene fusion, we believe that the RovA protein is also less abundant in the *hfq* mutant. Indeed products of three RovA-dependent genes are deregulated in our strain, i.e. decreased amount of RovA-activated InvA, and increased amounts of RovA-repressed OmpX and tryptophanase TnaA^17^,^37^. This constitutes the first report of a link between Hfq and RovA in enteropathogenic yersiniae. In *Y*. *pestis* and in *S*. Typhimurium, contrary to *Y*. *enterocolitica*, microarray analysis showed an increase in *rovA*/*slyA* transcript in an *hfq* mutant^29^,^32^, suggesting different evolutionary paths for Hfq-mediated regulation. In *Y*. *enterocolitica*, through its effect on OmpR, Hfq is likely to affect many bacterial properties associated with the bacterial surface in addition to InvA production, such as flagellation, serum resistance and *yadA* expression^20^,^22^,^23^.

### Complex role of Hfq in *yadA* expression

**Figure 3:**
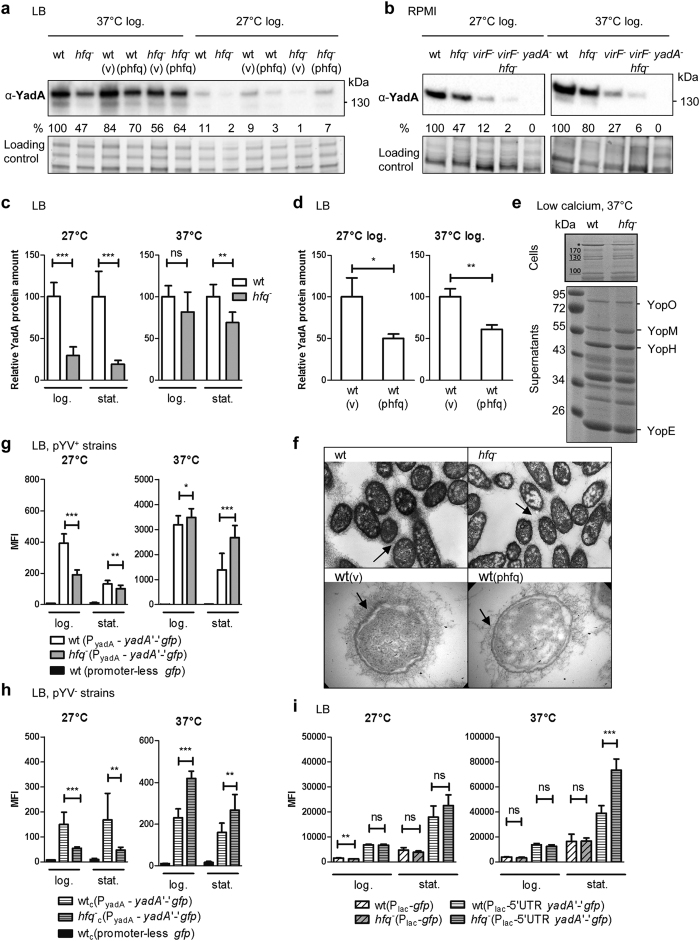
Complex regulation of adhesin YadA by Hfq. (**a–f**) Absence of *hfq*, but also multiple copies of *hfq*, lead to decreased YadA abundance. (**a**) Immunodetection of YadA in *Y. enterocolitica* strain WA-314 and derivatives grown in LB for 4 h. Upper panel shows the immunoblot and bottom panel shows part of the gel stained for total proteins. Percentages indicate the relative YadA signal intensities. (**b**) Hfq promotes production of YadA, independently of the VirF transcriptional activator. Strain WA-314 and derivatives were grown in RPMI for 4 h. (**c**,**d**) Semi-quantitative analysis of total YadA protein amounts in parent and *hfq* mutant grown in LB for 4h (log.) or 20 h (stat.). Results are the relative mean signal and standard deviation obtained from six (**c**) or three (**d**) independent cultures loaded on the same gel. (**e**) Hfq is dispensable for T3SS but not YadA production. Coomassie blue-stained gels of cell extracts (top) and supernatants (bottom) prepared upon growth in T3SS-inducing conditions. The asterisk indicates the YadA trimer. (**f**) Hfq impacts deposition of the collagen fibrils associated to YadA at the bacterial surface. Electron microscope images of bacteria grown for 20 h at 37 °C in collagen gels. Original magnification: upper panels, ×15000; lower panels, ×21000. Arrows point to the YadA-dependent deposited collagen fibrils. (g and h) Hfq exerts opposite effects on *yadA* expression at 27 °C and 37 °C (**g**), also in the absence of the pYV plasmid (**h**). Fluorescence of strain WA-314 and derivatives carrying promoter-less *gfp* or the translational fusion *yadA*‘-’*gfp* under the control of promoter P_yadA_ was measured by flow cytometry upon growth in LB for 4 h and 22 h. (**i**) Hfq inhibits *yadA* expression at the post-transcriptional level at 37 °C in stationary phase. Bacterial MFI of strains carrying pFX-1 (P_lac_–driven *gfp*) and pFX-Plac-yadA (*yadA* 5′UTR and *yadA*‘-’ *gfp* fusion under control of P_lac_). Results are the MFI and standard deviation of at least two independent experiments, each with three independent cultures per strain. Significance was calculated with Student’s unpaired *t*-test (***P ≤ 0.001; **P ≤ 0.01, *P ≤ 0.05; ns, not significant, *P* > 0.05).

Whereas InvA is the key adhesin for transcytosis of M-cells, the major adhesin for survival in deeper tissue is the trimeric autotransporter YadA^2^,^3^. Immunoblotting revealed that Hfq modulates YadA production (Fig. 3a–d). When grown in LB, RPMI or in T3SS-inducing conditions, the *hfq*-negative strain produced less YadA than the parental strain (Fig. 3a,b,e). This phenotype was strongest in LB at 27 °C with a 5-fold decrease that was complemented with phfq (Fig. 3a,c). Thus, at suboptimal temperature, Hfq facilitates YadA production. At 37 °C, Hfq appeared to play a role mainly in stationary phase, when YadA was less abundant in *hfq* mutants (ca. 40% decrease) (Fig. 3c). Interestingly, although *yadA* and *yop* genes share the same transcriptional activator VirF^14^, the production and secretion of Yop effector proteins is unaffected by a mutation in *hfq*^37^ (Fig. 3e), suggesting that Hfq promotes production of YadA independently of pYV-encoded VirF. Accordingly, in a *virF* mutant background, a mutation in *hfq* also leads to a decrease in YadA (Fig. 3b). At first glance, our results suggest that Hfq participates in the positive regulation of YadA production. However, our complementation analyses hints that the link between YadA and the RNA chaperone might be more complex. Indeed wild type carrying multiple copies of *hfq* (on plasmid phfq) produced less YadA than those with the control vector (Fig. 3d), suggesting that an optimal concentration of Hfq is required to control the amount of YadA adhesin. The complex effect of Hfq on YadA was also observed in bacteria grown in three-dimensional collagen gels that mimic host tissue environment^45^. In this model, YadA-producing bacteria grow in packed microcolonies reminiscent of microabscesses seen in tissue^45^ and the interaction of YadA with collagen results in a fibrillar matrix surrounding the bacteria^46^. Electron microscopy showed that *hfq* mutants, but also strains carrying multiple copies of *hfq*, were coated with sparser YadA-collagen fibrils than the parent (Fig. 3f), suggesting that the amount of Hfq needs to be tightly controlled for optimal YadA production also in a tissue-like environment. Taken together, our results indicate that Hfq promotes YadA production, independently of VirF, but that under some conditions, Hfq might also act as a repressor of YadA production.

As the next step in understanding the significance of Hfq for YadA regulation, we studied the expression of *yadA* using plasmid pFX-yadA, which carries the promoter, 5′ UTR and the first 16 codons of *yadA* fused in frame with *gfp*. Fluorescence of the *hfq* mutant grown at 27 °C was reduced 2-fold relative to the parental strain in exponential phase, also in the absence of pYV (Fig. 3g,h). Therefore, consistent with the results obtained by immunoblotting, Hfq promotes expression of *yadA* at 27 °C, independently of VirF. At 37 °C, we observed relatively minor differences in exponential phase (Fig. 3g) but surprisingly in stationary phase we detected a 2-fold increase in the MFI of the *hfq* mutant compared to parent, also in strains lacking pYV (Fig. 3g,h). These observations suggest that Hfq participates in repression of *yadA* expression at 37 °C in stationary phase. Next, we performed Northern blotting, but despite repeated attempts, we were unable to detect *yadA* mRNA in wild-type and *hfq*-negative strains grown at 37 °C (data not shown), suggesting that the *yadA* transcript is particularly unstable or expressed at low levels. Indeed to detect *yadA* in the parental strain by RT-qPCR we had to increase the total number of cycles from 40 to 50 and observed that the *yadA* mRNA increased by 10- to 430 fold in the *hfq* mutant relative to the parent in three biological replicates. These data are in agreement with the results obtained with reporter strains at 37 °C, but the variability was too high to reach statistical significance. Taken together, our results suggest that Hfq inhibits *yadA* expression at 37 °C in stationary phase. To test whether Hfq-mediated repression occurred post-transcriptionally, we generated plasmid pFX-Plac-yadA, where the promoter of *yadA* was replaced by the P_lac_ promoter. As shown in Fig. 3i, the MFI of the *hfq*-negative mutant carrying pFX-Plac-yadA increased by 70% compared to the parent in stationary phase at 37 °C. Therefore, we conclude that Hfq is able to inhibit *yadA* expression at the post-transcriptional level in stationary phase. In exponential phase, however, Hfq did not seem to play a major role in post-transcriptional regulation of *yadA* expression (Fig. 3i).

Our analysis revealed a discrepancy between gene expression (increase in *yadA*‘-’*gfp* expression and mRNA levels) and protein production (decrease of YadA protein detected by immunoblotting) at 37 °C in stationary phase. We believe that YadA might be processed post-translationally by an Hfq-dependent protease. Indeed YadA is known to be subjected to degradation by the periplasmic protease DegP^47^ and our previous proteomic analysis showed an increase in DegP in the *hfq* mutant^37^. Future work will clarify whether Hfq modulates stability of the YadA protein or alternatively that of the full length *yadA* mRNA transcript. Nevertheless, our study uncovered that Hfq can mediate post-transcriptional inhibition of *yadA* in stationary phase at 37 °C and constitutes the first description of this level of control for an adhesin secreted by the type V system. This finding is potentially significant as YadA belongs to a family of autotransporters involved in pathogenesis in several bacteria^48^. We suppose that the Hfq-dependent control exerted at the transcriptional (at 27 °C), post-transcriptional (at 37 °C in stationary phase) and likely post-translational (37 °C in stationary phase) levels is involved in fine-tuning the amount of YadA present at the bacterial surface in response to different environments.

### Hfq-dependent repression of Myf fimbriae

**Figure 4:**
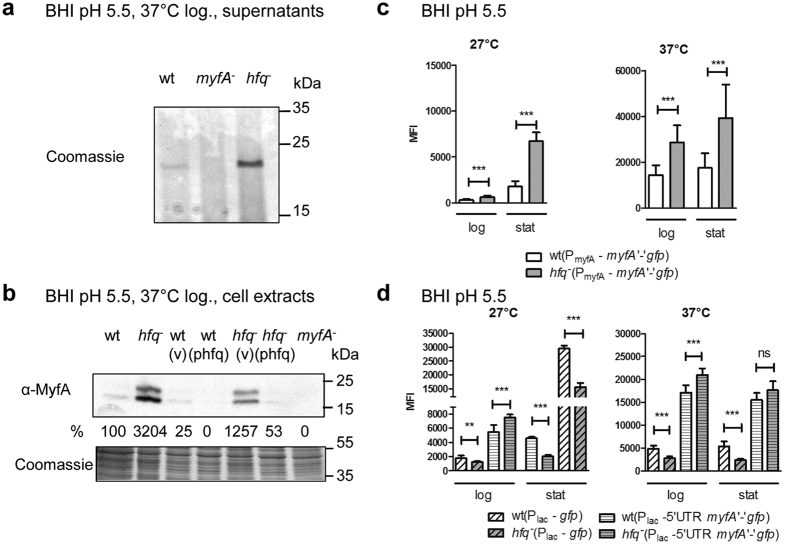
Hfq-dependent inhibition of MyfA pilin production (**a,b**) and *myfA* expression (**c,d**). (**a**,**b**) Production of MyfA pilin subunit by *Y*. *enterocolitica* strain JB580v and derivatives grown at 37 °C in BHI pH 5.5 for 5 h. (**a**) Coomassie-blue stained gel showing proteins released into the supernatants. (**b**) Immunodetection of MyfA in total cell extracts. Upper panel is the immunoblot and bottom panel shows part of the Coomassie blue-stained gel used as loading control. In the total extracts, MyfA is detected as two bands (24 and 20  Da) that likely represent the prepilin and pilin after cleavage of its signal peptide, respectively, and were both used for quantification. The normalized signal for MyfA relative to wild type (which was set to 100%) is indicated. (v), vector pACYC184ts; (phfq), plasmid phfq. **(c,d)** MFI of strain JB580v and *hfq* mutant carrying: (**c**) pFX-myfA (translational fusion *myfA*‘-’*gfp* under control of P_myfA_ promoter), and (**d**) pFX-1 (*gfp* under control of P_lac_) or pFX-plac-myfA (*myfA* 5′UTR and *myfA*‘-’*gfp* fusion under control of P_lac_) (**d**). Results are the MFI and standard deviation of at least two independent experiments, each with three independent cultures per strain. Significance was calculated with Student’s unpaired *t*-test (***P ≤ 0.001; **P ≤ 0.01; *P ≤ 0.05; ns, not significant, *P* > 0.05). In these experiments, the MFI of strains carrying plasmid pFX-0 was comprised between 7 and 23 units.

After uncovering the role of Hfq in the expression of non-fimbrial adhesins in *Y*. *enterocolitica*, we turned our attention to a fimbrial adhesin, the Myf fimbriae which is assembled by the chaperone/usher pathway. In *Y*. *enterocolitica*, the 21-kDa pilin subunit MyfA is expressed upon growth at 37 °C at acidic pH and is the major protein detected in supernatants of bacterial cultures under these conditions^15^. Analysis of supernatants and total extracts revealed that MyfA is produced in much higher amounts in the *hfq* mutant than its isogenic parent (ca. 32-fold increase), a phenotype that was reversed with plasmid phfq (Fig. 4a,b). Thus, our data shows that Hfq represses the production of Myf fibrillae. Next, to assess whether Hfq influenced expression of *myfA*, we generated plasmid pFX-myfA, which carries a translational fusion of the first 7 codons of *myfA* with *gfp* under the control of the P_myfA_ promoter. When grown in LB or in BHI at neutral pH, Hfq was largely dispensable for *myfA* expression (Supplementary Fig. S2). In striking contrast, upon growth in acidified BHI medium, we observed a 2- to 4-fold increase in MFI of the *hfq* mutant compared to the parent (Fig. 4c), indicating that Hfq inhibits expression of *myfA*. Under growth in acidified BHI, we detected comparable amounts of Hfq protein than upon growth at pH 7.2 (Supplementary Fig. S2), suggesting that one or more pH-dependent co-factor(s) are likely to play a role in the Hfq-dependent regulation of *myfA*. To test if the repression was post-transcriptional, we used pFX-Plac-myfA where the P_lac_ promoter drives the expression of the 5′ UTR of *myfA* and the *myfA*-*gfp* translational fusion. Control strains carrying plasmid pFX-1 revealed that the promoter activity of P_lac_ is reduced up to 55% in the *hfq*-negative strain (Fig. 4d). Results with strains carrying pFX-Plac-myfA indicated that Hfq exerts both an effect at the transcriptional or post-transcriptional level, depending on the conditions tested. First, in stationary phase at 27 °C, the *hfq* mutant carrying pFX-Plac-myfA exhibited a similar 50% decrease in MFI to that of strains with pFX-1 (Fig. 4d), suggesting that the Hfq-dependent repression of *myfA* expression is mainly at the transcriptional level. Second, especially in log phase at either 27 °C or 37 °C, the *hfq* mutant carrying pFX-Plac-myfA showed an increased MFI despite the reduced P_lac_ promoter activity (Fig. 4d). Taken together, these data suggest that Hfq inhibits *myfA* expression transcriptionally in stationary phase at 27 °C and post-transcriptionally during log phase at both temperatures.

In *Yersinia* spp. transcription of *myfA*/*psaA* is under the control of two membrane components MyfE/PsaE and MyfF/PsaF^49^,^50^ but has only been integrated in the RovA regulon in *Y*. *pestis* and not in *Y*. *enterocolitica*^17^. Interestingly our study also uncovered differences between *Yersinia* spp. regarding Hfq-dependent regulation of *myfA*/*psaA*: upon growth in standard LB medium, a strain lacking *hfq* expressed more *psaA* and *rovA* in *Y*. *pestis*^32^ but not in *Y*. *enterocolitica*. These differences might reflect the adaptation of these species to different lifestyles. In *Y*. *pestis* and *Y*. *pseudotuberculosis*, post-transcriptional regulation of *psaA* had long been hypothesized, based on (i) detection of *psaA* mRNA but no PsaA protein at 28 °C and low pH and (ii) on its unusually long 5′ UTR^50^,^51^. Our study implicates Hfq in the transcriptional and also post-transcriptional repression of *myfA* in *Y*. *enterocolitica* upon growth at low pH. Therefore, we speculate that Hfq-dependent sRNAs regulating *myfA* expression might be synthesized mainly at low pH during exponential phase.

### Increased production and length of O-Ag in *hfq*-negative strains

**Figure 5:**
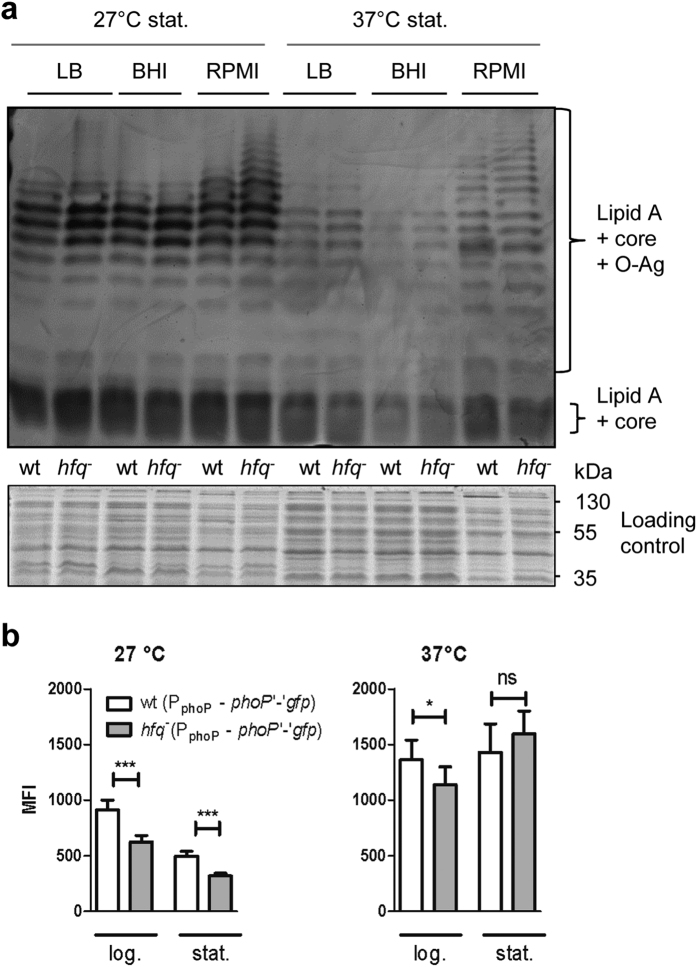
Hfq inhibits production and elongation of the O-Ag (**a**) and promotes expression of the regulatory gene *phoP* at 27 °C (**b**). **(a)** Top panel shows LPS silver staining and bottom panel shows loading controls with Coomassie blue-stained proteins prior to proteinase K digests. Bacteria were grown for 24 h in LB, BHI and RPMI at 27 °C and 37 °C. Loading was as follows: odd lanes, parental strain JB580v; even lanes, *hfq*-negative strain SOR17. **(b**) Fluorescence of parental strain JB580v and its isogenic *hfq* mutant carrying a translational fusion of *phoP* with *gfp* was measured by flow cytometry. Bacteria were grown in LB for 4 h (log.) and 22 h (stat.). Results are the MFI of at least two independent experiments, each with three independent cultures per strain. Significance was calculated with Student’s unpaired *t*-test (***P ≤ 0.001; *P ≤ 0.05; ns, not significant, *P* > 0.05).

With the discovery that many OMPs are regulated by the RNA chaperone Hfq, we turned our attention to LPS, another surface molecule associated with pathogenicity^6^,^7^,^8^,^9^,^24^. We previously reported that loss of Hfq is associated with increased production of LpxR, a LPS-modifying enzyme^37^. Using silver staining, we detected an increase in the amount and length of the O-Ag in the *hfq* mutant, the latter especially upon growth in RPMI (Figs 5 and S3). These differences could be complemented with plasmid phfq (Fig. S3). Interestingly, introduction of multiple copies of *hfq* into the parent inhibited production of LPS with long O-Ag at 37 °C (Supplementary Fig. S3). Thus, Hfq represses the production of LPS with long O-Ag in *Y. enterocolitica* under certain growth conditions. In agreement with our finding Hfq downregulates production of proteins involved in O-Ag synthesis in *S*. Typhimurium^29^. In *Y. enterocolitica* both *rovA* and *phoP* negatively regulate the expression of *lpxR* and positively control expression of loci necessary for LPS modifications with aminoarabinose and palmitate^8^,^24^. Having previously established a slight positive effect of Hfq on *rovA* expression (Fig. 2d), we next tested whether expression of *phoP* was also influenced by *hfq*. Using a translational fusion of the first five codons of *phoP* with *gfp* (under control of the *phoP* promoter), we observed that Hfq modestly promotes expression of *phoP* at 27 °C (Fig. 5b). Our observation contrasts with reports in *E*. *coli* and *S*. Typhimurium of an inhibitory effect of Hfq on *phoP* expression^29^,^52^, suggesting distinct rewiring of regulatory networks among enterobacteria. Thus we have now observed that Hfq positively impacts expression of both regulators *rovA* and *phoP*, albeit modestly. These results correlate well with the increase in the *rovA*- and *phoP*-repressed LpxR protein detected in the *hfq* mutant^37^ and suggest that Hfq profoundly impacts the lipid composition of the outer membrane in *Y. enterocolitica*. The RNA chaperone might participate in the adaptation of LPS to the host temperature, leading to a less immunogenic LPS and thus a dampened recognition by the innate immune system.

### Reduced translocation of YopH into host cells by *hfq* mutants

**Figure 6:**
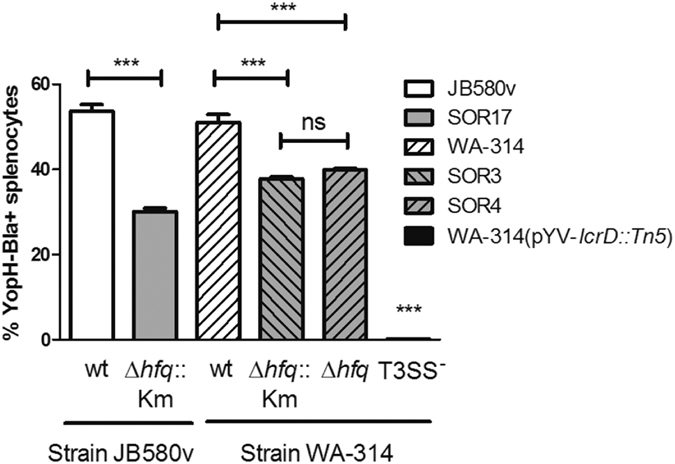
Hfq promotes translocation of YopH into splenocytes. Bacterial strains carrying a YopH-β-lactamase fusion were grown for 90 min at 37 °C in BHI prior to infection of isolated mice splenocytes at a MOI of 20. After 75 min of infection, cells were stained with the green fluorescent substrate CCF4-AM for 90 min. Blue fluorescence (resulting from substrate cleavage by injected YopH-Bla) and green fluorescence of splenocytes were measured by flow cytometry. Results represent the mean percentage of blue cells and standard deviation of duplicate infections and are representative of two independent experiments. Significance was calculated with One-way ANOVA (*P* < 0.001) with post-hoc Bonferroni’s Multiple Comparison test (***P ≤ 0.001; ns, not significant *P* > 0.05). The CFUs in the inoculum were determined by plating and were as follows: JB580v, 1.7 × 10^7^; SOR17, 1.9 × 10^7^; WA-314, 3.9 × 10^7^; SOR3, 7.2 × 10^7^; SOR4, 5.9 × 10^7^.

With two major adhesins YadA and InvA less abundant in *hfq*-negative strains, we expected that interaction of *Y*. *enterocolitica* with host cells would be affected. Moreover with increased length of the O-Ag, the short adhesin Ail, even if more abundant, would be poorly exposed^2^ and unlikely to substitute for downregulated YadA and InvA. Both YadA and InvA mediate tight adhesion to and signaling in host cells that facilitate translocation of Yop effectors by the T3SS^53^. Therefore, we tested the translocation of YopH-Bla (fusion of YopH with the reporter enzyme β-lactamase) into isolated mice splenocytes with the CCF4-AM substrate, which does not stain bacteria but penetrates into eukaryotic cells. Injected YopH-Bla converts the green fluorescence of CCF4-AM into blue fluorescence due to its β-lactamase activity^54^. As a negative control we used a T3SS mutant which is unable to translocate Yop proteins (Fig. 6). Although all bacterial strains produced similar amounts of YopH-Bla prior to infection (Supplementary Fig. S4), the percentage of blue cells was reduced by 20–45% for the three independent *hfq* mutants tested compared to their parental strains (Fig. 6), indicating that Hfq promotes translocation of YopH-Bla into host cells.

### Attenuated virulence of *hfq* mutant in mice

**Figure 7:**
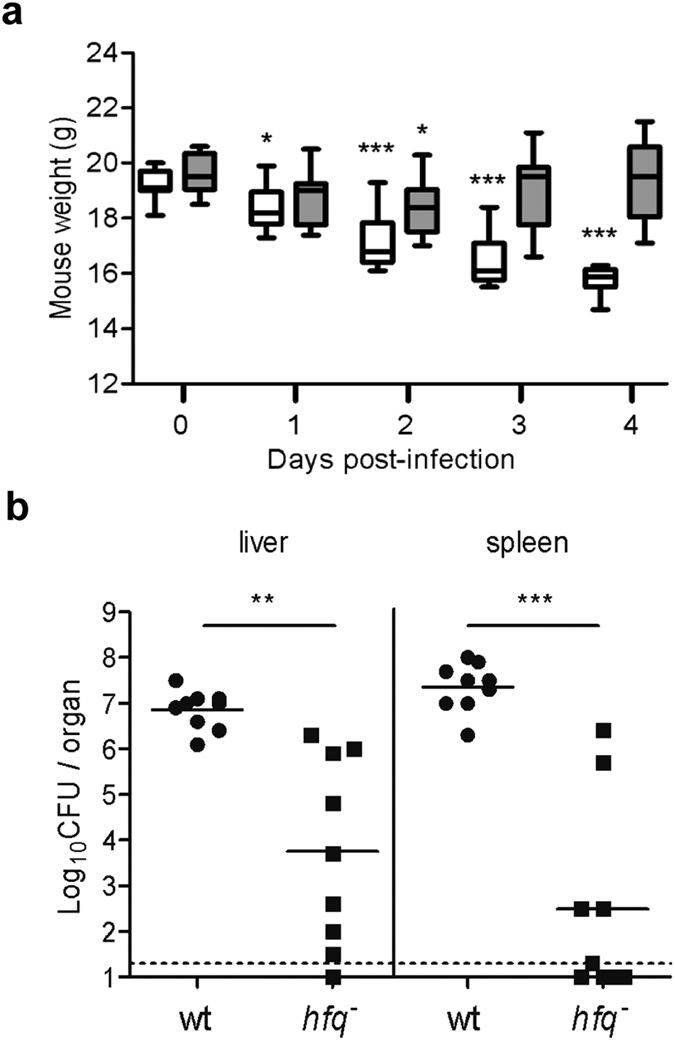
Hfq is required for virulence of *Y*. *enterocolitica* in Balb/c mice. Balb/c mice (*n* = 9 per group) were infected intraperitoneally with 4 × 10^3^ CFUs of *Y*. *enterocolitica*. (**a**) Mice body weight during infection with parent strain WA-314 (white bars) and *hfq*-negative strain SOR4 (grey bars). Median weight is indicated by a horizontal line, the boxes show the 25^th^ and 75^th^ percentile and the whiskers show the range. Significance was calculated with Student’s unpaired *t*-test between day 0 and the subsequent time points (*P < 0.05, ***P < 0.001). (**b**) Number of CFUs recovered from liver and spleen after four days of infection with parent (black circles) and *hfq* mutant (black squares). The limit of detection is indicated by a dashed line, and symbols below represent mice that did not have detectable numbers of bacteria. A solid line represents the mean of CFUs recovered. Significance was calculated using the unpaired *t t*est with Welch’s correction (**P < 0.005, ***P < 0.0005).

To test the importance of *hfq* for *Y*. *enterocolitica* virulence, we infected Balb/c mice intraperitoneally with parent strain WA-314 or *hfq* mutant SOR4 and determined the bacterial load in the liver and spleen five days post-inoculation. Mice infected with the *hfq* mutant showed fewer disease symptoms including less weight loss as those infected with the wild type (Fig. 7a). After five days of infection, recovery of *hfq*-negative bacteria was reduced 1,000- and 10,000- fold from liver and spleen, respectively, compared to the parent (Fig. 7b). Attenuation of the *hfq* mutant in mice likely reflects a combination of factors, such as an increased susceptibility to oxidative and acid stress, a difference in metabolism and disruption in the customary regulation of virulence factors (ref. 37 and this study).

### Overall conclusions

**Figure 8:**
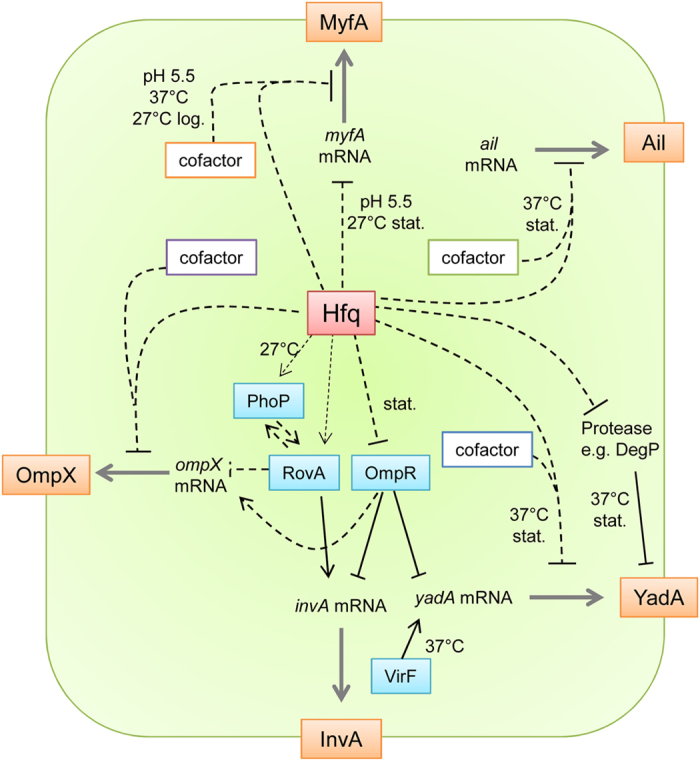
Working model for the role of Hfq in the regulation of adhesins in *Y*. *enterocolitica*. The production of adhesins (in orange) is controlled by the RNA chaperone Hfq through its influence on transcriptional regulators (in blue) and through post-transcriptional inhibition (action on the grey arrows). We hypothesize that Hfq acts in concert with different cofactors (boxed), likely sRNAs, to exert its post-transcriptional effects on the different adhesins. Hfq represses expression of *myfA* (only when bacteria are grown at low pH) at the post-transcriptional level in logarithmic phase and through an effect on *myfA* transcription at 27 °C (in stationary phase). Production of Ail is controlled by Hfq at the post-transcriptional level at 37 °C. Hfq exerts a complex influence on YadA production, independently of VirF, the major direct transcriptional activator of *yadA*: (i) At 27 °C, Hfq promotes expression of *yadA*, possibly at the transcriptional level through its effect on the direct transcriptional repressor OmpR; (ii) conversely with the help of an unknown cofactor produced at 37 °C in stationary phase, Hfq represses *yadA* post-transcriptionally, (iii) ultimately, through its impact on proteases, like the periplasmic DegP, Hfq might also control YadA protein stability. Hfq controls the expression *ompR* and *rovA* which encode two of the four DNA-binding proteins that directly regulate *invA*. Finally, production of OmpX is negatively controlled by Hfq through its concerted effects on the transcriptional regulators RovA and OmpR and on the post-transcriptional level in coherent feed-forward loops. Black lines represent a reported direct regulation, such as the direct binding of a transcriptional regulator of the gene promoter (e.g. RovA and OmpR on the promoter region of *invA*) or the direct interaction of YadA with the protease DegP.

In this study, we demonstrated the importance of the RNA chaperone Hfq for the virulence of *Y*. *enterocolitica*. Among the most striking phenotypes associated with mutations in *hfq* was the differential production of surface pathogenicity factors, i.e. the non-fimbrial adhesins Ail, InvA and YadA, the fimbrial adhesin Myf and LPS. Although two previous studies have highlighted the role of Hfq in the virulence of *Y*. *pestis* and *Y*. *pseudotuberculosis*^32^,^36^, no reports have so far uncovered the profound impact of the RNA chaperone on adhesins and LPS in pathogenic yersiniae. Our study suggests that the effects of Hfq are likely to be direct (by post-transcriptional control of mRNAs encoding Ail, YadA and MyfA) and/or indirect (by modulating regulatory genes that contribute to remodelling the bacterial surface, i.e. *rovA*, *ompR* and *phoP*) (see model, Fig. 8). Interestingly, not all the envelope pathogenicity factors are impacted by Hfq, e.g. T3SS remains functional for protein secretion *in vitro*^37^. However, since both major adhesins YadA and InvA are downregulated in *hfq*-negative strains, interaction of *Y. enterocolitica* with host cells is likely to be modified. Indeed we observed a decrease in protein translocation by the T3SS, consistent with this hypothesis.

Hfq typically acts in conjunction with sRNAs to modulate mRNA stability and/or translation^27^. Using translational fusions with *gfp* under the control of the exogenous promoters, we could show that at least part of the Hfq-dependent repression of *ompX*, *ail*, *yadA* and *myfA* is post-transcriptional. We have previously shown that Hfq production increases during growth of *Y. enterocolitica* and is maximal in late exponential and stationary phases at 27 °C and 37 °C^37^. It has also been reported that sRNAs are most abundant in stationary phase in *Y. pseudotuberculosis*^31^. In this study, stationary phase coincided likewise with maximal Hfq-dependent repression for *ompX*, *ail* and *yadA*. Still, post-transcriptional repression by Hfq was not uniform for all adhesins: for *myfA*, repression occurred in BHI at pH 5.5, but not in LB, and for *ail*, inhibition ensued at 37 °C but not at 27 °C (Table S4). These differences suggest the involvement of distinct cofactors in addition to Hfq in controlling synthesis of the different adhesins (Fig. 8). Hfq might stabilize distinct sRNAs or regulate alternate RNA-binding proteins. Future work will aim to identify Hfq cofactors mediating post-transcriptional regulation. Taken together, our results suggest that Hfq participates in a complex interplay of transcriptional, post-transcriptional and post-translational processes involved in fine-tuning components of the bacterial envelope.

## Methods

### Ethics statement

All animal work was performed in strict accordance with the German regulations of the Society for Laboratory Animal Science (GV-SOLAS) and the European Health Law of the Federation of Laboratory Animal Science Associations (FELASA). The protocol was approved by the Regierung von Oberbayern, Sachgebiet 54 (Verbraucherschutz und Veterinärwesen). All efforts were made to minimize suffering.

### Bacterial strains and media

*Y*. *enterocolitica* biotype 1B, serotype O:8 and *E*. *coli* strains used in this study are listed in Table S1. Bacteria were routinely grown in LB broth (10 g tryptone, 5 g yeast extract and 5 g NaCl per liter) and on LB-agar at 27 °C (*Y*. *enterocolitica*) and 37 °C (*E*. *coli*). SOC medium (0.5% yeast extract, 2% tryptone,10 mM NaCl, 2.5 mM KCl, 10 mM MgCl2, 10 mM MgSO4, 20 mM glucose) was used to recover bacteria after electroporation and prior to plating. For production of the Myf fimbriae, *Y*. *enterocolitica* strains were grown in Porcine Brain Heart Infusion (BD, Heidelberg) supplemented with 0.5% yeast extract and adjusted to pH 5.5. For maximal production of YadA, bacteria were also grown in RPMI 1640 without phenol red (Life Technologies, Darmstadt). Antibiotics were used at the following concentration: ampicillin (Ap), 100 μg/ml (for *E. coli*); carbenicillin (Cb), 300 μg/ml (for *Y*. *enterocolitica*); chloramphenicol (Cm), 20 μg/ml; kanamycin (Km), 50 μg/ml; spectinomycin (Sp), 100 μg/ml for strain JB580v and its derivatives, and 200 μg ml^−1^ for strain WA-314 and its derivatives. For blue-white screening of colonies, LB agar was supplemented with IPTG (4.8 μg/ml, Carl Roth) and X-Gal (4 μg/ml, Carl Roth).

### Protein staining and immunoblotting

Protein extracts from comparable optical density (OD) equivalents were denatured at 95 °C for 5 min, chilled on ice, separated by SDS-PAGE (Mini-Protean Tetra-cell, Bio-Rad, Munich) and stained with Coomassie brilliant blue. For semi-quantitative immunoblotting, proteins were separated on Bio-Rad Stain-free precast gels and subsequently reacted with the trihalo compound within the gels by a 2.5-min exposure to ultraviolet light using a ChemiDoc MP imager (Bio-Rad), allowing quantification of total proteins loaded per lane. Proteins were transferred to Roti-PVDF membranes (cat. T830.1, Carl Roth, Karlsruhe) using a Semi-Dry-Blotter (Carl Roth) according to manufacturer’s instructions. Immunoblots were reacted with antibodies as previously described^37^. Chemiluminescence detection was performed using Immobilon Western Chemiluminescent HRP substrate (Merck Millipore, Darmstadt) and the ChemiDoc MP imager (Bio-Rad). Images were analysed with the “Lane and Bands” tool of ImageLab 4.1 software (Bio-Rad). For each lane, we determined the density of the bands detected by chemiluminescence (on the immunoblot) and the intensity of stain-free fluorescence (in the gel prior to blotting). For background subtraction, a rolling disc value of 10 was applied. To adjust for differences in loading, the “Normalization” tool of ImageLab software was used to normalize the chemiluminescent signal using the stain-free fluorescence of all proteins in the corresponding lane^55^. Primary antibodies used were rabbit polyclonal antibodies directed against OmpX from *E*. *coli* (1:5,000, a kind gift from D. Linke, Tübingen), InvA (1:2,000), MyfA (1:2,000)^16^, YopH (1:5000) or YadA (1:5000)^56^. We also used mouse monoclonal antibodies directed against YadA (8D1, diluted 1:1,000)^57^, Ail (3C6, 1:2,000, a kind gift from J. Bliska, Stony Brook, NY) and the FLAG epitope (anti-FLAG M2, 1:2000, Sigma). Secondary antibodies were horseradish peroxidase-conjugated anti-rabbit or anti-mouse immunoglobulin G (GE Healthcare), both diluted 1:20,000.

### Molecular biology

Purification of bacterial genomic DNA, plasmid DNA, PCR products and DNA from agarose gels was performed using the corresponding GeneJet kits from Thermo Scientific. Routine PCR was performed with RedTaq enzyme mix (VWR) whereas GoldTaq (Roche) or HighFidelity Enzyme Mix (Thermo Scientific) were used for cloning purposes. DNA oligonucleotide primers were from Biomers.net (Ulm, Germany) and are listed in Table S2. DNA sequencing was performed by LGC genomics (Berlin, Germany). DNA sequence analysis was performed with the software package Lasergene 8 (DNASTAR).

### RNA isolation and quality assessment

*Y*. *enterocolitica* strains were grown in 30 ml LB overnight at 27 °C shaking at 175 rpm in an Excella E24 incubator (New Brunswick Scientific), diluted in 50 ml fresh LB to OD = 0.1 and grown for 4 h at 37 °C shaking at 200 rpm in a Certomat BS-1 incubator (B. Braun Biotech International). Following OD measurements, a sample containing 5 × 10^8^ CFUs was mixed with two volumes of RNAprotect Bacteria Reagent (Qiagen, Hilden, Germany), incubated at room temperature (RT) for 5 min, before centrifugation for 10 min at 5000 × g. After decanting the supernatant, pellets were stored at −20 °C between 6 and 20 days. For RNA preparation, pellets were then thawed at RT and mixed by vortexing with 100 μl lysozyme solution (1 mg/ml lysozyme in 10 mM Tris-Cl, 1 mM EDTA, pH 8.0) and incubated at RT for 5 min in a shaker-incubator (Thermomixer, Eppendorf). Bacterial lysate was then mixed 700 μl QIAzol Lysis Reagent from the miRNeasy Mini (Qiagen). The RNA isolation proceeded as the manufacturer’s recommendation and included an on-column DNase digestion (Qiagen) with a final elution in 50 μl RNase-free water. Successful DNA digestion was confirmed by failure to amplify DNA in a conventional PCR with 35 cycles using rRNA gene-specific primers OR154-Y.16S-86f and OR155-Y.e.ame.16S-455r. Following measurements with a spectrophotometer (Nanodrop ND-1000, Peqlab Biotechnologie), RNA concentrations ranged between 385 and 580 ng/μl, A_260_/A_280_ ratios were comprised between 2.0 and 2.1 and A_260_/A_230_ ratios were generally above 1.7. RNA integrity was assessed using the Experion Automated Electrophoresis system, stdSens RNA chip and reagents (Bio-Rad) and gave RQI (RNA quality indicator) numbers comprised between 8.7 and 9.5.

### Northern blotting

Northern blots were performed using the DIG Northern Starter Kit and the DIG Wash and Block Buffer Set (Roche) according to manufacturer’s instructions. Luminescence signal detection was performed with the ChemiDoc MP imager (Bio-Rad). For each sample, one μg RNA was separated by electrophoresis in MOPS/formaldehyde gel. A digoxigenin (DIG)-labeled RNA molecular weight marker (Roche) was also included on the gel. For RNA probe preparations, DNA templates were first amplified by PCR, using primers OR237 and OR278 for *ompX*, OR238 and OR277 for *ail*, and primers OR189 and OR281 for *yadA*. The DNA template was subsequently used in an *in vitro* transcription reaction with DIG-labeled nucleotides and T7 RNA polymerase according to manufacturer’s instructions.

### Generation of translational fusions with GFP

To generate translational fusions with the green fluorescent protein in the pFX-P plasmid^43^, we used the Golden Gate cloning technique^58^. DNA fragments carrying promoter, 5′ untranslated region (5′-UTR) and the first 4–16 codons of a gene of interest were amplified by PCR. In most constructs at least 320 bp upstream of the published transcriptional start were included, except *myfA* (74 bp) and *ompR* (156 bp). The primers used for this purpose are listed in Table S2 and contain *Bsa*I sites and additional sequences designed to generate compatible ends with *Bsa*I-digested pFX-P. In a 20-μl Golden Gate cloning reaction, 40 fmol of vector were mixed with 40 fmol of PCR product, 5 units of *Bsa*I enzyme (New England Biolabs, Frankfurt) and 4.5 units of ligase (Thermo Scientific) in ligase buffer, incubated for 1 h at 37 °C, then 5 min at 50 °C followed by 5 min at 80 °C, and electroporated into *E. coli*. White clones were selected on LB agar supplemented with Sp, IPTG and X-Gal and confirmed by PCR (using primer OR178 and the forward primer specific for the cloned fragment) and by plasmid DNA sequencing (using primer OR177 or OR178). We also constructed fusions where the endogenous gene promoter was replaced by the P_lac_ promoter. For this a 115-bp PCR fragment containing P_lac_ was amplified by PCR from plasmid pFX-P using primers OR181 and OR182. This product was mixed together in a Golden Gate cloning reaction with pFX-P and a DNA fragment amplified by PCR which contained the 5′-UTR and the first 4–16 codons of the gene of interest.

To generate plasmid pFX-2, primers OR239 and OR235 were used to amplify a 201-bp fragment containing P_LtetO-1_ from plasmid pXG10-SF^59^ by PCR, and the fragment was cloned into pFX-P in a Golden Gate cloning reaction. To construct pFX-PtetO-ail, an 114-bp DNA fragment containing the 5′ UTR and first nine codons of *ail* was amplified by PCR using primers OR160 and OR238 and genomic DNA from strain JB580v as a template. This fragment was mixed together in a Golden Gate reaction with pFX-P and a 131-bp PCR fragment containing P_LtetO-1_ amplified with primers OR235 and OR236 from the template plasmid pXG10-SF. Plasmids used in this study are listed in Table 1.

### Generation of YopH-reporter

The *yopH* gene (including the promoter and coding sequence of *yopH* without the stop codon) was amplified from pYV_WA-314_ plasmid using the forward primerYopHprom-HindIII-F containing HindIII site, and the reverse primer YopH/Stop-AflII-B containing AflII site. TEM-1 β-lactamase gene (*bla*) was amplified from the plasmid pBR322 using the forward primer (G_4_S)_3_Bla-AflII-F containing AflII site and a sequence coding for the linker (L) peptide (GGGGS)_3_, and the reverse primer Bla-SalI-B containing SalI site. The PCR products containing *yopH* and *bla* were digested with HindIII/AflII and AflII/SalI, respectively, and ligated, together in one step, into HindIII/SalI site of the low-copy plasmid pACYC184 to generate plasmid pYopH-L-Bla.

### Flow cytometry

Flow cytometry was used to measure the fluorescence of individual bacteria carrying pFX-P plasmid derivatives. For this, single bacterial colonies grown on agar plates were subcultured overnight in 2 ml LB supplemented with Sp. Three independent overnight cultures were used for each strain, diluted 1:20 in 2 ml fresh media and subsequently grown at 27 °C or 37 °C. After 4 h and after ca. 22 h, the bacteria were diluted in sterile phosphate-buffered saline (PBS) to ca. 4–8 × 10^6^ CFU/ml. Fluorescence (using the FITC filter settings) of at least 20,000 events per sample was acquired with the FACS Canto II (BD Biosciences, Heidelberg, Germany) and analyzed with the FACS Diva Software v6.1.2.

### Supernatant analysis

To induce secretion by the pYV-encoded T3SS, bacteria were grown for 90 min at 37 °C in LB supplemented with 5 mM EGTA and 0.2% glucose. To test the production of Myf fibrillae, bacteria were grown at 37 °C for 2 or 5 h in BHI supplemented with 0.5% yeast extract and adjusted to pH 5.5. Cultures were centrifuged at 2,600 × g for 10 min at 4 °C, and supernatants were precipitated as previously described^37^.

### Growth in three-dimensional collagen gels

Bacteria were grown in three- dimensional collagen gels (3D-CoG) as described previously^45^. For electron microscopy, samples were fixed in 1.17% glutaraldehyde, 0.03% ruthenium red in 0.2 M sodium cacodylate buffer (pH 7.3) for 1 h at RT, followed by three washes in 0.1 M sodium cacodylate buffer (pH 7.3). Samples were then incubated for 3 h in 1.67% osmium tetraoxide, 0.5% ruthenium red in 0.2 M sodium cacodylate buffer (pH 7.3) at RT, then washed with 0.1 M sodium cacodylate buffer (pH 7.3) at RT before being dehydrated in a graded series of ethanol (30, 50, 70, 90, and 100%). Embedding was performed in araldite with polymerization for 48 h at 60 °C, followed by thin sectioning and staining with unbuffered uranyl acetate according to standard procedures. The stained samples were imaged in a Philips CM-10 electron microscope.

### T3SS Translocation assay

For translocation assays, splenocytes were prepared from mice spleens: the isolated organs were pressed with a sterile syringe plunger in sterile cold PBS first through a 70-μm, then a 40-μm cell strainer. Following centrifugation at 300 × g for 5 min, cells were resuspended in erythrocyte-lysis buffer (8.29 g NH_4_Cl, 0.783 g NH_4_HCO_3_, 0.0371 g EDTA per liter), incubated for 10 min at RT and centrifuged again. After resuspension in RPMI 1640 (Life Technologies), cells were aliquoted in microtiter plates with 1 × 10^6^ cells/well, were then infected with *Y*. *enterocolitica* strains at a multiplicity of infection (MOI) of 20. After 75 min of infection, the cells were transferred into a microfuge tube and resuspended in 1 × CCF4-AM staining solution supplemented with probenecid, prepared according to the manufacturer’s instructions (Invitrogen, Carlsbad, CA). Cells were incubated at 29 °C for 90 min (protected from light) prior to fixation with 3.7% formaldehyde for 15 min at 4 °C. After two washes in FACS buffer (2% fetal calf serum, 0.09% sodium azide in PBS), cells were resuspended in FACS-buffer. Green and blue fluorescence of at least 100,000 cells/sample was analyzed by flow cytometry on a FACS Canto II (BD Biosciences) using FACS Diva software.

### Mouse infections

Six- to eight-week-old female Balb/c mice (Harlan, Winkelmann, Germany) were infected with 4 × 10^3^ CFU of *Y*. *enterocolitica* intraperitoneally. To prepare the inoculum, bacteria were grown overnight in BHI at 27 °C, diluted 1/40 in fresh BHI and cultured for 90 min at 37 °C. The inoculum was subsequently prepared by washing and diluting the bacteria in sterile cold PBS. Mice were weighed and visually inspected daily. They were sacrificed four days after infection by CO_2_ asphyxiation. Liver and spleen were aseptically removed and homogenized with a bead mill (MM2000, Retsch, Haan) in 3- and 1-ml PBS, respectively, and bacterial counts per organ were determined by plating serial dilutions on LB plates.

### Statistical analyses

Statistical analyses were performed using the Prism 5 software (v. 5.01, GraphPad).

## Additional Information

**How to cite this article**: Kakoschke, T. K. *et al*. The RNA Chaperone Hfq Is Essential for Virulence and Modulates the Expression of Four Adhesins in *Yersinia enterocolitica*. *Sci. Rep.* **6**, 29275; doi: 10.1038/srep29275 (2016).

## Supplementary Material

Supplementary Information

## Figures and Tables

**Table 1:** Plasmids used in this study.

Plasmid name	Promoter/gene fusion	Description	Source or Reference
pACYC184		origin p15A (15 copies per cell), Cm^R^, Tetracycline^R^	New England Biolabs
pACYC184ts		origin p15A (15 copies per cell), Cm^R^, with a deletion in the tetracycline resistance gene	37
phfq		pACYC184-derivative carrying *Y*. *enterocolitica hfq*	37
pYopH-L-Bla		pACYC184 derivative carrying the promoter and coding sequence of the *yopH* gene (without stop codon) fused in frame through linker (L) peptide (GGGGS)_3_ to TEM-1 β-lactamase gene *bla*, Cm^R^	This study
pFX-P		origin RSF1010 (10-12 copies per cell), Sp^R^, *gfp* gene without start codon, used to generate translational fusions	43
pFX-0	Promoter-less /*gfp*	pFX-P carrying a promoter-less complete *gfp* gene, used as a negative control, Sp^R^	43
pFX-1	P_lac_/*gfp*	pFX-P carrying a complete *gfp* gene under control of the P_lac_ promoter	43
pFX-2	P_LtetO-1_/*gfp*	pFX-P carrying a complete *gfp* gene under control of the P_LtetO-1_ promoter	This study
pFX-ail	P_ail_ /*ail*‘-’*gfp*	pFX-P carrying 380 bp upstream of *ail* start codon, and 9 codons of *ail* fused in frame with *gfp*	This study
pFX-PtetO-ail	P_LtetO-1_/*ail*‘-’*gfp*	pFX-P carrying the P_LtetO-1_ promoter, 59 bp upstream of *ail* start codon, and 9 codons of *ail* fused in frame with *gfp*	This study
pFX-ompX	P_ompX_/*ompX*‘-’*gfp*	pFX-P carrying 402 bp upstream of *ompX* start codon, and 5 codons of *ompX* fused in frame with *gfp*	This study
pFX-Plac-ompX	P_lac_/*ompX*‘-’*gfp*	pFX-P carrying the P_lac_ promoter, 44 bp upstream of *ompX* start codon, and 5 codons of *ompX* fused in frame with *gfp*	This study
pFX-invA	P_invA_/*invA*‘-’*gfp*	pFX-P carrying 494 bp upstream of *invA* start codon, and 4 codons of *invA* fused in frame with *gfp*	This study
pFX-Plac-invA	P_lac_ /*invA*‘-’*gfp*	pFX-P carrying the P_lac_ promoter, 121 bp upstream of *invA* start codon, and 4 codons of *invA* fused in frame with *gfp*	This study
pFX-yadA	P_yadA_/*yadA*‘-’*gfp*	pFX-P carrying 686 bp upstream of *yadA* start codon, and 16 codons of *yadA* fused in frame with *gfp*	22
pFX-Plac-yadA	P_lac_/*yadA*‘-’*gfp*	pFX-P carrying the P_lac_ promoter, 264 bp upstream of *yadA* start codon, and 16 codons of *yadA* fused in frame with *gfp*	This study
pFX-rovA	P_rovA_/*rovA*‘-’*gfp*	pFX-P carrying 634 bp upstream of *rovA* start codon, and 6 codons of *rovA* fused in frame with *gfp*	This study
pFX-ompR	P_ompR_/*ompR*‘-’*gfp*	pFX-P carrying 188 nucleotides upstream of *ompR* start codon, and 4 codons of *ompR* fused in frame with *gfp*	This study
pFX-phoP	P_phoP_/*phoP*‘-’*gfp*	pFX-P carrying 860 bp upstream of *phoP* start codon, and 5 codons of *phoP* fused in frame with *gfp*	This study
pFX-myfA	P_myfA_/*myfA*‘-’*gfp*	pFX-P carrying 173 bp upstream of *myfA* start codon, and 7 codons of *myfA* fused in frame with *gfp*	This study
pFX-plac-myfA	P_lac_/*myfA*‘-’*gfp*	pFX-P carrying the P_lac_ promoter, 99 bp upstream of *myfA* start codon, and 7 codons of *myfA* fused in frame with *gfp*	This study
