# Supplementary material for: The RNA Chaperone Hfq Is Essential for Virulence and Modulates the Expression of Four Adhesins in Yersinia enterocolitica
Source: Sci Rep. 2016 Jul 8;6:29275. doi: 10.1038/srep29275 (PMC4937351; doi:10.1038/srep29275)
Supplement: Supplementary Information [file srep29275-s1.pdf]

Supplementary material

The RNA chaperone Hfq is essential for virulence and modulates the expression of four adhesins in *Yersinia enterocolitica*

Tamara Katharina Kakoschke[1], Sara Carina Kakoschke[1], Catharina Zeuzem[1], Hicham Bouabe[1], Kristin Adler[1], Jürgen Heesemann[1] and Ombeline Rossier[1*]

[1] Max von Pettenkofer Institute for Hygiene and Medical Microbiology, Ludwig Maximilians University, Munich, Germany

* Corresponding author

Supplementary Methods

Bacterial strains

Y. *enterocolitica* biotype 1B, serotype O:8 and *E*. *coli* strains used in this study are listed in Table S1.

RT-qPCR

RNA was isolated from three cultures grown on separate occasions (three biological replicates per strain). cDNA synthesis was performed in duplicate for each RNA sample with the Maxima First strand cDNA synthesis kit (cat. K1652, ThermoFischer Scientific, St. Leon-Rot, Germany) according to manufacturer's instructions, using 1 µg RNA, 100 pmol random hexamer primers and 1µl M-MuLV-derived reverse transcriptase in a 20-µl reaction. Control reactions without reverse transcriptase were also performed in parallel (RT-minus control). Prior to addition of the enzyme, RNAs were denatured for 5 min at 65°C. Reactions were incubated for 10 min at 25°C, then 30 min at 50°C, followed by 5 min at 85°C. cDNAs and diluted cDNAs were stored at -80°C for 2-10 weeks prior to qPCR. cDNAs were diluted 1/10 in RNase-free water containing 100 ng/µl tRNA (cat. 10 109 495 001, Roche).

qPCR was performed using FastStart Essential DNA Green Master Mix (cat. 06402712001, Roche, Mannheim, Germany) according to manufacturer's instructions. Primers specific for *ail*, *ompX* and 16S rRNA genes were designed using Primer Blast (http://www.ncbi.nlm.nih.gov/tools/primers-blast/) and the strain sequence of *Y*. *enterocolitica* strain 8081 (NC_008800.1) (Table S2, Table S3). The total reaction volume was 25 µl, including 5 µl of template (diluted cDNA) and 2.5 µl of primer mix at the appropriate concentrations (Table S3). PCR runs were performed with a Lightcycler 96 Instrument (Roche) with the following settings: initial denaturation at 95°C for 10 min, 40 cycles of denaturation at 95°C for 15 s, annealing at 60°C for 30 s, and elongation at 72°C for 30 s. After each run, a melting curve analysis was performed to confirm specificity. RT-minus control and reactions without template had quantification cycles (C_q) higher than 36, confirming absence of DNA contamination and specificity of the reaction. For each primer pair, standard curve and thus reaction efficiency were determined with dilution series of genomic DNA from strain JB580v (diluted in water containing 100 ng/µl tRNA). For the standard curves, reactions were performed in triplicates (PCR replicates) and analyzed using LightCycler 96 Software Version 1.1 (Roche). Reaction efficiencies appear in Table S3. Each cDNA was analyzed in duplicate (PCR replicates), and the duplicate C_q values were averaged before calculating the relative expression levels of the gene of interest (*ail* or *ompX*) in the *hfq*-negative strain, normalized to 16S rRNA gene, compared to the wild-type strain. Expression ratios (R) were calculated with the following equation: $R=(1+E_{gene})^{\Delta Cq(gene)} / (1+E_{16S\ rRNA})^{\Delta Cq(16S\ rRNA)}$, where E is the PCR reaction efficiency and $\Delta Cq=C_q(wt)-C_q(hfq)$. The resulting expression ratios were averaged for cDNA replicates (duplicate reactions with reverse transcriptase).

Table S1. Strains used in this study

Strains	Description	Source or Reference
Y. enterocolitica		
WA-314	Clinical isolate of serotype O:8, carrying virulence plasmid $pYV_{WA\text{-}314}$	[1]
WA-C	$pYV_{WA\text{-}314}$-cured derivative of WA-314	[1]
WA-C-inv	WA-C derivative, *invA*::Km, invasin-negative	[2]
WA-314 myfA	WA-314 derivative, *myfA*::Sp defective in Myf fibrillae production	[3]
WA-314(pYV-A-0)	WA-314 derivative, *yadA*::Km defective in YadA production	[4]
WA-314(pYVΔvirF)	WA-314 derivative carrying pYVΔ*virF*::Km, reduced YadA and Yop production	[5]
WA-314(pYV-515)	WA-314 derivative carrying pYV-*lcrD*::Tn5, defective in Yop secretion	[2]
SOR3	WA-314 derivative with a deletion of *hfq* marked with a Km^R cassette	[6]
SOR4	WA-314 derivative with an unmarked deletion of *hfq*	[6]
SOR5	$pYV_{WA\text{-}314}$-cured WA-314 derivative with an unmarked deletion of *hfq*	[6]
SOR5(pYVΔvirF)	SOR5 carrying pYVΔ*virF*::Km	This study
JB580v	Derivative of clinical isolate 8081, restriction endonuclease-negative (R^-), methyltransferase-positive (M+), carrying virulence plasmid pYV_{8081}	[7]
JB580c	pYV_{8081}-cured derivative of JB580v	This study
SOR17	JB580v derivative with a deletion of *hfq* marked with a Km^R cassette	[6]
SOR17c	pYV_{8081}-cured SOR17 derivative	This study
SOR35	JB580v derivative with an unmarked chromosomal fusion of *hfq* with sequences encoding the 3xFLAG epitope	[6]
8081v-Ye2v	8081v derivative, defective in Ail production	[8]
8081-2428-c	YeO8-*wzz*-GB	[9]
8081-R2	Rough derivative of 8081-R^- M^+	[10]
E. coli		
DH5α	*fhuA2 lac(del)U169 phoA glnV44* Phi80*lacZ(del)M15 gyrA96 recA1 relA1 endA1 thi-1 hsdR17*	Invitrogen, Darmstadt

Table S2. Primers used in this study

Primer name	Sequence	Used for
OR160-ail-pFX-rv	TTTGGTCTCTTAGCAGAACTAGCTAGTAATGTC	pFX-cloning
OR161-ail-pFX-fw	TTTGGTCTCTATTCGCATGATAGATGAGCTTCAG	pFX-cloning
OR162-ompX-pFX-rv	TTTGGTCTCTTAGCTGCAATTTTATTCATAACCAC	pFX-cloning
OR163-ompX-pFX-fw	TTTGGTCTCTATTCCCTGTTGAGCCTCAAAG	pFX-cloning
OR164-yadA-pFX-rv	TTTGGTCTCTTAGCCGCAGATATTAATGCCGCAG	pFX-cloning
OR166-yadA-pFX-fw	TTTGGTCTCTATTCCCGTTTATGGTTCCAGACA	pFX-cloning
OR177	CCATGCTCAGAAAAGGCTTAACA	pFX sequencing
OR178	CCGTATGTAGCATCACCTTCA	pFX sequencing
OR179-invApFX-fw	TTTGGTCTCTATTCTGAAGGAGCAACATATAGG	pFX-cloning
OR180-invApFX-rv	TTTGGTCTCTTAGCAAATGAATACATTAGTGTACC	pFX-cloning
OR181-plac-fw	TTTGGTCTCTATTCTGAGCGCAACGCAATTAATG	pFX-cloning
OR182-plac-rv	TTTGGTCTCTCCACCCACACAACATACGAGCCGG	pFX-cloning
OR183-invA-pFXplac-fw	TTTGGTCTCTGTGGATTGTTAGTGTTTGCGAGAG	pFX-cloning
OR184-rovA-pFXfw	TTTGGTCTCTATTCAGCCTCCAGTGCTTACCAAC	pFX-cloning
OR185-rovA-pFXrv	TTTGGTCTCTTAGCTCCTAATGTCGATTCCAATTGC	pFX-cloning
OR186-rovA-pFXplac-fw	TTTGGTCTCTGTGGTGTGTTTTTAATGTTAATTAC	pFX-cloning
OR189-yadA-pFXplac-fw	TTTGGTCTCTGTGGTAACACTTTTCGTGTTATCTGA	pFX-cloning
OR190-ompX-pFXplac-fw	TTTGGTCTCTGTGGGGTGTTTTAGTTTCACTT	pFX-cloning
OR204-myfA-pFX-rv	TTTGGTCTCTTAGCAACAAATTTTTTCATATTC	pFX-cloning
OR205-myfA-pFX-fw	TTTGGTCTCTATTCTGCGTCAGTATTTATTAAAT	pFX-cloning
OR208-phoP-pFX-rv	TTGGTCTCTTAGCAACTAAAACCCGCATACTAC	pFX-cloning
OR209-phoP-pFX-fw	TTTGGTCTCTATTCGGCAAATCAACGCCACCTGA	pFX-cloning
OR210-ompR-pFX-rv	TTTGGTCTCTTAGCATTCTCTTGCATTTTATTAC	pFX-cloning
OR211-ompR-pFX-fw	TTTGGTCTCTATTCATGGTGAGGCGACCTACTG	pFX-cloning
OR235-PtetO-F	TTTGGTCTCTATTCTAAGAAACCATTATTATC	pFX-cloning
OR236-PtetO-R	TTTGGTCTCTGTGCTCAGTATCTCTATCA	pFX-cloning
OR238-pFX-PtetO-ail-F	TTTGGTCTCTGCACAGCCATGTCAGTGATATGGT	pFX-cloning
OR239-pFX-2	TTTGGTCTCTTAGCCATAGCTGTTTCCTGTG	pFX-cloning
OR264-pFX-plac-myfA-F	TTTGGTCTCTGTGGCGTCAGTGATGACACCG	pFX-cloning
OR154-Y.16S-86f	GCGGCAGCGGGAAGTAGTTTA	qPCR
OR155-Y.e.ame.16S-455r	CAATCCAACAACGTATTAAGTTATTGG	qPCR
OR63-ail-RT-F	GCCTGTTTATCAATTGCGTCTGT	qPCR
OR64-ail-RT-R	TGGCTTTGCGCATAACCAA	qPCR
OR71-yadA-RT-F	TGCCCCATAAGTAACTGCCG	qPCR
OR72-yadA-RT-R	GTGCTGAAGCAGCGAAACAA	qPCR
OR187-16S-RT-F	CCCCCTGGACAAAGACTGAC	qPCR
OR188-16S-RT-R	TCAAGGGCACAACCTCCAAG	qPCR
OR197-ompX-RT-F	TACGGATGCGACTCTGTTCG	qPCR
OR198-ompX-RT-R	ATGGTGCGGGTATGCAGTTT	qPCR
OR277-T7prom-ail-R	TAATACGACTCACTATAGGGCTGCACCAAGCATC	Northern
OR278-T7prom-ompX-R	TAATACGACTCACTATAGGGGAAAGTGTAACCAAC	Northern
OR281-T7prom-yadA-R	TAATACGACTCACTATAGGGGTTATTTGCGATCC	Northern
YopHprom-HindIII-F	CGCAAGCTTCTTATGGGAAAAGCCAAAAAACTAACGAACAC	YopH-Bla cloning
YopH/Stop-AflII-B	CGCCTTAAGGCTATTTAATAATGGTCGCCCTTGTCCTTC	YopH-Bla cloning
(G4S)3Bla-AflII-F	CGCCTTAAGGGTGGAGGCGGTTCAGGCGGAGGTGGCAGCGGCGGT GGCGGATCGCTACTTACTCTAGCTTCCCGGCAAC	YopH-Bla cloning
Bla-SalI-B	CGCGTCGACTTACCAATGCTTAATCAGTGAGGC	YopH-Bla cloning

Table S3. Primer name and concentration, amplicon size and reaction efficiency for qPCR analysis of target gene expression

Target gene	Primer pair	Primer concentration	Amplicon size	Reaction efficiency
ail	OR63/OR64	62.5 nM	77 bp	0.93
ompX	OR197/OR198	125 nM	75 bp	1.01
yadA	OR71/OR72	250 nM	125 bp	0.98
16S rRNA	OR187/OR188	50 nM	111 bp	1.10

Table S4. Summary of Hfq-mediated regulation in *Y. enterocolitica* stain JB580v using translational fusions with *gfp*[a]

	**27°C**		**37°C**	
**Gene fused with *gfp***	Log.	Stat.	Log.	Stat.
ompX	neg.[b], pt[c]	neg., pt	neg., pt	neg., pt
ail	no[d]	no	neg., pt	neg., pt
invA	pos.[e]	pos.	pos.	pos.
yadA	pos.	no	no	neg., pt
myfA	no	no	no	no
***myfA* (BHI pH 5.5)**	neg., pt	neg.	neg., pt	neg., pt
rovA	pos.	pos.	pos.	pos.
ompR	neg.	neg.	neg.	neg.
phoP	pos.	pos.	pos.	no

[a]Regulation tested upon growth in LB unless stated otherwise in parenthesis
[b]neg.: negatively regulated by Hfq
[c]pt: post-transcriptional regulation
[d]no: no influence of Hfq
[e]pos.: positively regulated by Hfq

Supplementary References

1. Heesemann, J. and R. Laufs, *Construction of a mobilizable Yersinia enterocolitica virulence plasmid.* J Bacteriol, 1983. **155**(2): p. 761-7.
2. Ruckdeschel, K., et al., *Differential contribution of Yersinia enterocolitica virulence factors to evasion of microbicidal action of neutrophils.* Infect Immun, 1996. **64**(3): p. 724-33.
3. Trcek, J., et al., *Gut proteases target Yersinia invasin in vivo.* BMC Res Notes, 2011. **4**: p. 129.
4. Roggenkamp, A., et al., *Substitution of two histidine residues in YadA protein of Yersinia enterocolitica abrogates collagen binding, cell adherence and mouse virulence.* Mol Microbiol, 1995. **16**(6): p. 1207-19.
5. Freund, S., et al., *Unusual, virulence plasmid-dependent growth behavior of Yersinia enterocolitica in three-dimensional collagen gels.* J Bacteriol, 2008. **190**(12): p. 4111-20.
6. Kakoschke, T., et al., *The RNA chaperone Hfq impacts growth, metabolism and production of virulence factors in Yersinia enterocolitica.* PLoS One, 2014. **9**(1): p. e86113.
7. Kinder, S.A., et al., *Cloning of the YenI restriction endonuclease and methyltransferase from Yersinia enterocolitica serotype O8 and construction of a transformable R-M+ mutant.* Gene, 1993. **136**(1-2): p. 271-5.
8. Wachtel, M.R. and V.L. Miller, *In vitro and in vivo characterization of an ail mutant of Yersinia enterocolitica.* Infect Immun, 1995. **63**(7): p. 2541-8.
9. Bengoechea, J.A., et al., *Functional characterization of Gne (UDP-N-acetylglucosamine-4-epimerase), Wzz (chain length determinant), and Wzy (O-antigen polymerase) of Yersinia enterocolitica serotype O:8.* J Bacteriol, 2002. **184**(15): p. 4277-87.
10. Zhang, L., et al., *Molecular and chemical characterization of the lipopolysaccharide O-antigen and its role in the virulence of Yersinia enterocolitica serotype O:8.* Mol Microbiol, 1997. **23**(1): p. 63-76.

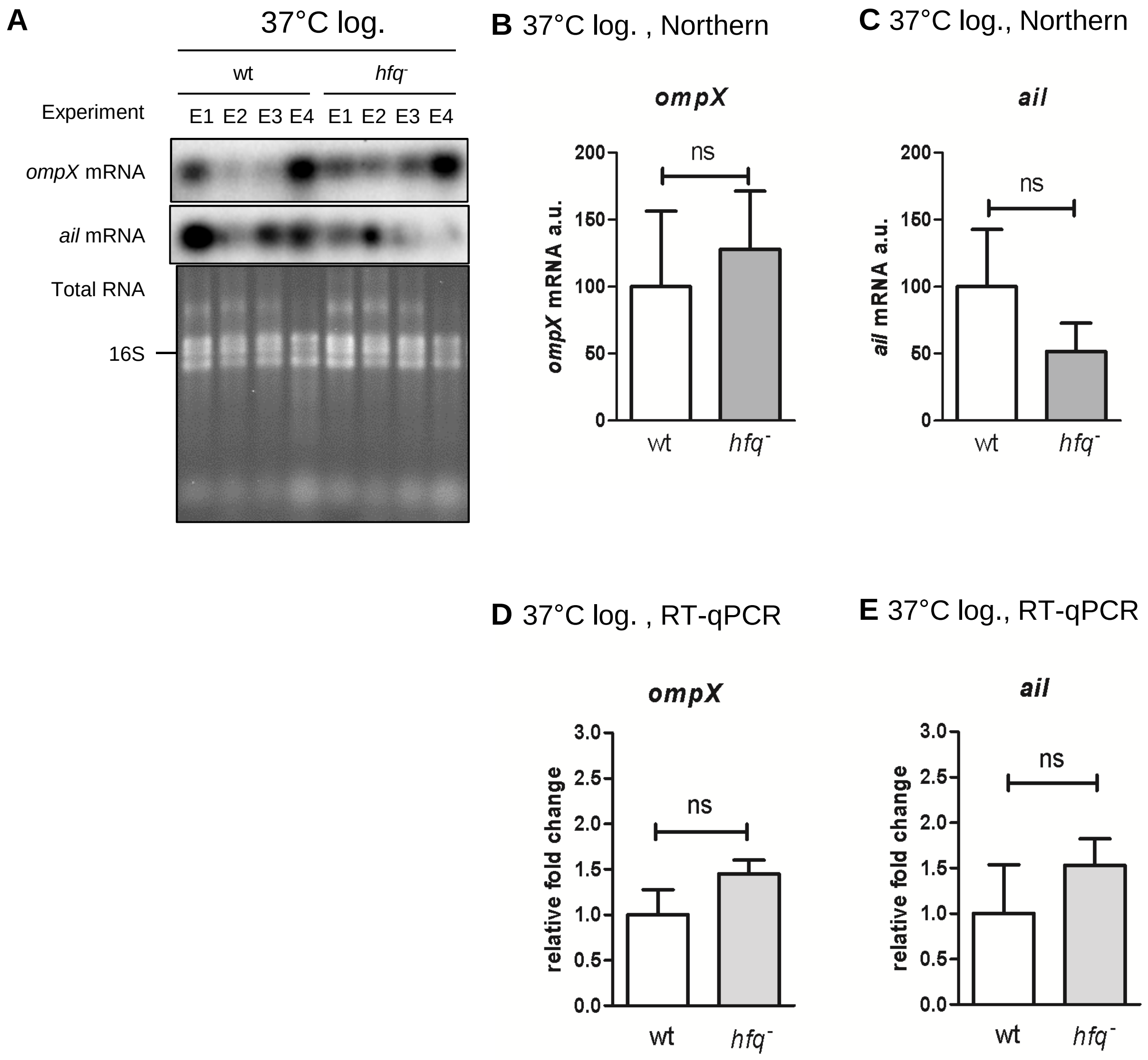

Fig. S1. Transcript levels of *ompX* and *ail* in *Y. enterocolitica* during logarithmic phase.
(A) Northern blotting with DIG-labelled *ompX*- or *ail*-specific RNA probe. Bacteria were grown on four separate occasions in LB at 37°C for 4 h (E1-E4) (log.). Upper two panels show Northern blots and bottom panel shows ethidium-bromide stained total RNA. Note that due to an intervening sequence, the 23S ribosomal RNA is processed into two RNA species that flank the 16S rRNA band. **(B and C)** Semi-quantitative analysis of *ompX* and *ail* mRNAs detected by Northern blotting in panel (A). (a.u.), arbitrary units. **(D and E)** RT-qPCR was used to determine the mean fold change in transcript abundance in the *hfq* mutant relative to parent (which was set at 1). RNA was isolated in separate experiments than for the Northern blots shown in panel A. Parental strain JB580v and *hfq*-negative strain SOR17 were grown for 4 h in LB at 37°C, and RNA was harvested from three independent cultures grown on separate occasions. Significance was calculated with Student's unpaired *t*-test (ns, not significant $P>0.05$).

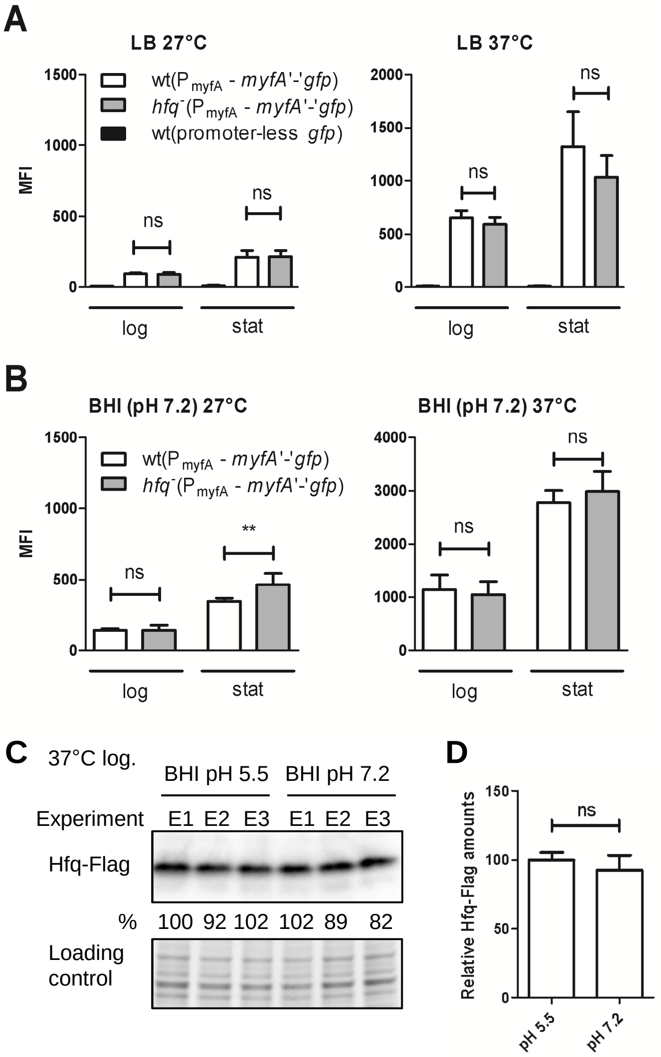

Fig. S2. Effect of Hfq on *myfA* expression upon growth in LB (A) or BHI pH 7.2 (B) and analysis of Hfq production upon growth in BHI at different pH (C and D). (A and B) Fluorescence of strains carrying plasmid pFX-0 (promoter-less *gfp*) or plasmid pFX-myfA (translational fusion of *myfA* with *gfp* under the control of the promoter P_{myfA}) was measured by flow cytometry. Bacteria were grown for 4 h (log.) and 22 h (stat.). Results are the MFI and standard deviation of two independent experiments, each with three independent cultures per strain. Significance was calculated with Student's unpaired *t*-test (** $P \leq 0.01$; ns, non significant $P > 0.05$). **(C)** Immunodetection of Hfq-Flag in total protein extracts of strain SOR35, a derivative of JB580v that carries a chromosomal fusion of *hfq* with sequences encoding the 3xFLAG epitope. Bacteria were grown at 37°C for 4 h in BHI at pH 5.5 or 7.2 on three separate occasions (experiments E1-E3). **(D)** Semi-quantitative analysis of immunoblot shown in (C).

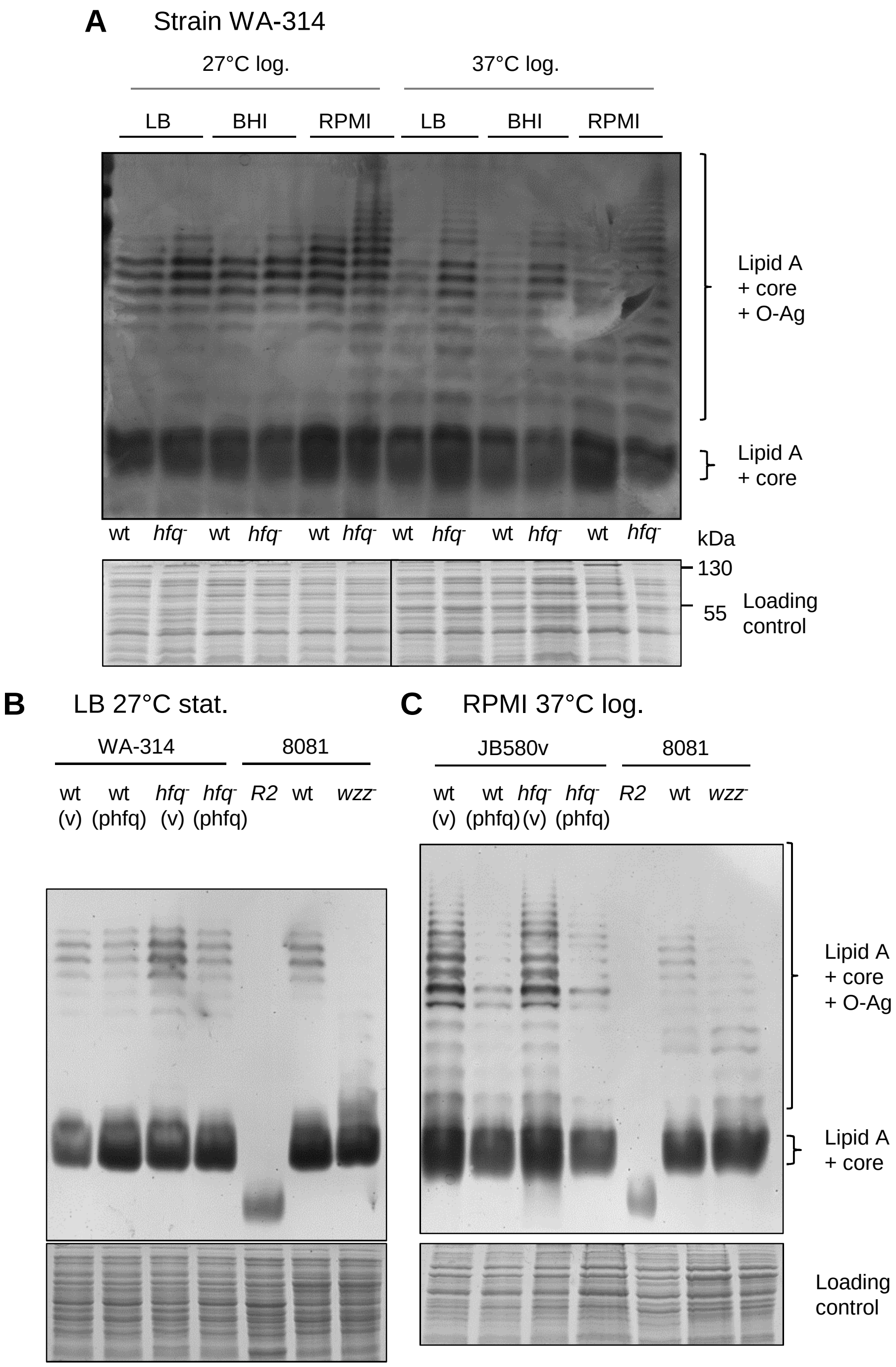

Fig. S3. LPS profile of *Y. enterocolitica* strains. Top panels show LPS silver staining and bottom panels show loading controls with Coomassie blue-stained proteins prior to proteinase K digests. (v), vector pACYC184; (phfq), pAhfq. **(A)** LPS silver staining of WA-314 parental strain and *hfq*-negative derivative SOR4. Bacteria were grown for 5 h in LB, BHI and RPMI at 27°C and 37°C. **(B)** Complementation analysis of *hfq* mutant SOR4. Bacteria were grown in LB at 27°C for 22 h. Loading was as follows: lane 1, WA-314(pACYC184); lane 2, WA-314(pAhfq); lane 3, SOR4(pACYC184); lane 4, SOR4(pAhfq); lane 5, rough mutant derivative of 8081 R2; lane 6, wild-type 8081; lane 7, O-Ag length determinant mutant 8081-2428-c wzz-GB. **(C)** Complementation analysis of *hfq* mutant SOR17. Bacteria were grown in RPMI at 37°C for 5 h. Loading was as follows: lane 1, JB580v(pACYC184); lane 2, JB580v(pAhfq); lane 3, SOR17(pACYC184); lane 4, SOR17(pAhfq); lane 5, rough mutant 8081-R2-c; lane 6, wild-type 8081; lane 7, O-Ag length determinant mutant 8081-2428-c *wzz*-GB.

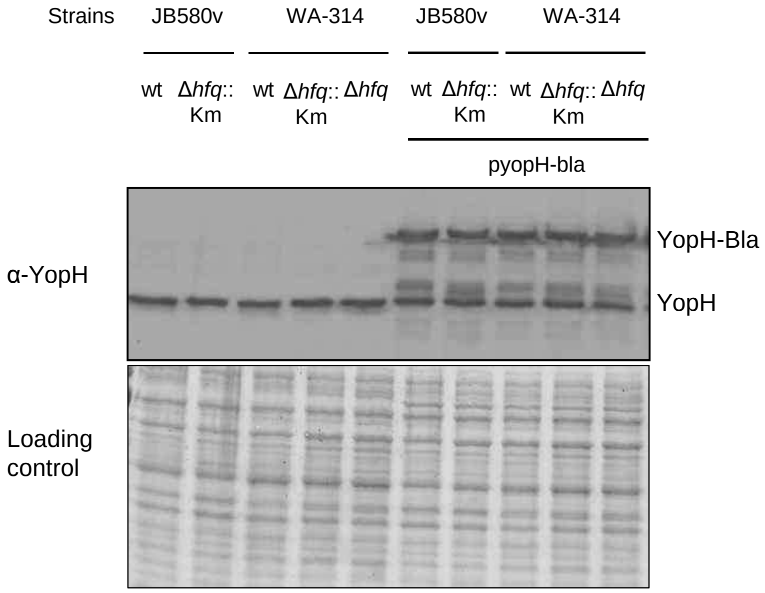

Fig. S4. Detection of YopH and YopH-Bla (YopH fused to β-lactamase) in total protein extracts of *Y. enterocolitica* grown in BHI at 37°C for 90 min, prior to infection of splenocytes. Top panel shows immunoblot, bottom panel shows part of the Coomassie-stained gel used as loading control. Loading was as follows: Lane 1, JB580v; 2, SOR17; 3, WA-314; 4, SOR3; 5, SOR4; 6, JB580v(pyopH-bla); 7, SOR17(pyopH-bla); 8, WA-314(pyopH-bla); 9, SOR3(pyopH-bla) and 10, SOR4(pyopH-bla).
